# Lineage-specific diversity of pheromone response pathway genes is independent of mating strategy in *Ceratocystidaceae*

**DOI:** 10.1186/s12864-026-12527-y

**Published:** 2026-02-23

**Authors:** Frances A. Lane, Brenda D. Wingfield, Michael J. Wingfield, P. Markus Wilken

**Affiliations:** Department of Biochemistry, Genetics and Microbiology, Forestry and Agricultural Biotechnology Institute (FABI), University of Pretoria, Private Bag X20, Hatfield, 0028 South Africa

**Keywords:** Signal transduction pathway, Mate communication, Sexual strategy, Taxonomic divergence, Evolutionary conservation, *Microascales*

## Abstract

The pheromone-receptor system plays a crucial role in mate recognition and fertilisation of ascomycete fungi and is strongly regulated by proteins coded for in the mating-type locus. This locus also determines the mating strategy, sexual identity and mate compatibility of fungal species. Genome assemblies are available for many species in the *Ceratocystidaceae*, a family of fungi with diverse mating strategies. This provided an opportunity to investigate the relationship between sexual strategy and the pheromone-receptor system in these species. The pheromone and pheromone-receptor genes were identified and characterised from the genome sequences of 52 isolates representing 35 species from 14 genera in the *Ceratocystidaceae*. Additionally, these data were used to elucidate patterns linked to differences between the pheromones and pheromone receptors, and mating strategies. To do this, the target genes were identified using homologous- and syntenic-based approaches. Putative protein sequences and genomic locations were then compared across the entire family, including species of *Huntiella* that had previously been characterised. With a few exceptions, putatively functional pheromones and receptors were identified in all the *Ceratocystidaceae* genomes. The results showed no discernible link between the presence, genomic location or structure of these proteins and mating strategy. Interspecific conservation in sequence and genomic locations revealed greater correlation to taxonomic relatedness, although intraspecific variation was also observed. Interestingly, despite reports of sexual reproduction in *Ambrosiella* species, all genomes of these species lacked the α-pheromone gene and encoded a non-functional α-pheromone receptor. This is the most extensive study of pheromones and their receptors in a *Sordariomycetes* family and complements efforts aimed at understanding the complex history of sexual reproduction in the *Ceratocystidaceae*.

**Supplementary Information:**

The online version contains supplementary material available at 10.1186/s12864-026-12527-y.

## Introduction

Ascomycete fungi exhibit remarkable diversity in both mating strategies and their underlying genetic architecture. Two primary mating behaviours are recognized in these fungi, known as homothallism (self-fertility) and heterothallism (self-sterility) [1]. These mating behaviours are governed by the genetic make-up of the mating-type locus (*MAT1*), which controls the expression of genes involved in sexual reproduction [2–4]. Heterothallic individuals have a bipolar mating system defined by two versions of the *MAT1* locus known as idiomorphs [5], with recombination possible only between individuals that differ in idiomorph type [1, 6]. Homothallism includes a complex assemblage of self-fertility mechanisms that differ in the content and structure of the mating-type locus [7, 8]. Of these, primary homothallism, pseudo-homothallism, unisexuality and mating-type switching are recognised mating strategies in homothallic filamentous ascomycetes [3, 9, 10].

The *MAT1* locus controls the functioning of the pheromone-receptor system [2, 11], a crucial facilitator of mating in ascomycetous fungi. The pheromone signalling pathway has been most extensively characterised in heterothallic species [12–14]. Individuals that have the *MAT1-1* idiomorph express the α-pheromone protein that, after modification, is secreted from the cell. Similarly, individuals with the *MAT1-2* idiomorph produce the a-pheromone protein that is further modified before being released. These pheromones are detected by one of two pheromone receptors, each specific to a pheromone type. The receptors are G-protein-linked, transmembrane proteins that are generally produced by all members of a species, regardless of mating specificity [15, 16]. When the pheromone secreted by one individual binds to the cognate pheromone receptor on the cell surface of a potential mating partner, it triggers a signal transduction pathway that results in mate recognition and begins the various steps of sexual reproduction [17, 18].

Functional studies in various filamentous ascomycetes have shown that the pheromone-receptor system has roles beyond mate recognition [11]. This is particularly evident in primary homothallic species, where mate recognition through the pheromone-receptor system is often unnecessary. While the system is not required for recombination in some of these species [19], in others it remains essential for selfing and outcrossing events [20–22]. For instance, in *Sordaria macrospora*, the deletion of pheromone and receptor genes can have varying effects on ascomata formation, including no effect, a drastic reduction, or complete prevention, depending on which genes and combination are deleted [21]. The ability to produce ascomata when parts of the pheromone-receptor system are absent suggests that this system enhances mating, rather than being essential for it [21, 22]. Additionally, the pheromone-receptor system is crucial for post-fertilisation events and is proposed to play a role in the interaction, fusion, and separation of nuclei [21].

Species of *Ceratocystidaceae* (*Microascales*) are best known for their association with plants and beetles, and have recently been shown to display a high diversity of mating strategies [23–25]. The genera *Berkeleyomyces*, *Ceratocystis*, *Endoconidiophora* and *Thielaviopsis* accommodate many important plant pathogens [26–29], commonly causing diseases in economically important crop plants and trees such as banana, cacao, eucalypts, mango, palms, spruce and sweet potato [23, 26, 27, 30–32]. Their ability to thrive in diverse environments, including plant tissues, water and soil, underscores their adaptability as pathogens. These fungi have also evolved a wide diversity of sexual reproductive strategies, many of which have been extensively characterised through full genome sequencing. These sexual strategies include heterothallism, primary homothallism, mating-type switching and unisexuality [9, 33–38].

Despite their important role in fungal reproduction, the pheromones and pheromone receptors of the *Ceratocystidaceae* have only been investigated in the genus, *Huntiella* [39, 40]. The aim of the present study was thus to identify and annotate the genes of the pheromone/receptor system in other *Ceratocystidaceae*, and subsequently to consider possible correlation with the mating strategies of these fungi. To achieve this goal, the genome assemblies of 54 isolates representing 37 species in 15 genera of the *Ceratocystidaceae* and related *Gondwanamycetaceae* were mined for genes involved in the pheromone/receptor pathway. Comparisons of the putative proteins encoded by the genes of the pheromone/receptor system, together with the positional conservation of each gene provided insights into the evolutionary history of this system in the *Ceratocystidaceae*.

## Materials and methods

### Genome assemblies

*Ceratocystidaceae* genome assemblies that were publicly available as of January 2024 were downloaded into a custom database for this study (Table 1). When available, this included multiple genome assemblies for a single species (e.g., *Berkeleyomyces rouxiae*, *Ceratocystis albifundus*, *Ce. fimbriata*, *Ce. manginecans* and *Thielaviopsis punctulata*) amounting to a total of 60 genome assemblies for 43 species residing in 14 genera. The genome sequences of the related species *Knoxdaviesia capensis* and *K. proteae* (*Microascales*; *Gondwanamycetaceae*) were also included as outgroup taxa (Table 1). The genome assemblies were assessed using QUAST (v. 5.3.0 + galaxy1; default settings with type of organism set to “Fungus”) [41] and BUSCO (v. 5.4.4; using sordariomycetes_odb10) [42] to ensure they were of sufficient quality for downstream analysis (Suppl. Table 1).

**Table 1:** Genome assemblies used in this study

Species	Isolate	Mating strategy	Accession Number	Genome Size (Mb)	Citation/NCBI Submitter
*Ambrosiella beaveri*	CBS 121753	Primary homothallic [43]	GCA_030294605	27.0	University of Pretoria
*A. cleistominuta*	CBS 141682	Primary homothallic [35, 43]	GCA_017139545	27.1	Wilken et al. 2020 [44]
*A. xylebori*	CBS 110.61	Primary homothallic [43]	GCA_002778035	27.2	Vanderpool et al. 2018 [45]
*Berkeleyomyces basicola*	CMW 49352	Heterothallic [33]	GCA_003671435	25.3	Wingfield et al. 2018 [46]
*Be. rouxiae*	PM34	Heterothallic [33]	GCA_026151695	28.2	Dumigan et al. 2023 [47]
	LT1	Heterothallic [33]	GCA_036245355 ^1^	25.5	Chiba University, Graduate School of Horticulture
*Bretziella fagacearum*	CMW 2656	Heterothallic [43]	GCA_002018255	26.8	Wingfield et al. 2016 [48]
*Catunica adiposa*	CBS 136.34	Mating-type switching [43]	GCA_001640685	28.3	Wingfield et al. 2016 [49]
*Ceratocystis albifundus*	CMW 4068	Mating-type switching [38]	GCA_002742255	29.3	van Der Nest et al. 2019 [50]
	CMW 13980	Mating-type switching [38]	GCA_002742155 ^1^	27.1	van Der Nest et al. 2019 [50]
	CMW 17274	Mating-type switching [38]	GCA_002742205 ^1^	26.4	van Der Nest et al. 2019 [50]
	CMW 17620	Mating-type switching [38]	GCA_000813685 ^1^	26.9	van Der Nest et al. 2014 [51]
	CMW 24680	Mating-type switching [38]	GCA_002742215 ^1^	26.3	van Der Nest et al. 2019 [50]
	CMW 24685	Mating-type switching [38]	GCA_002742195 ^1^	26.8	van Der Nest et al. 2019 [50]
	CMW 24860	Mating-type switching [38]	GCA_012650125 ^1^	33.1	University of Pretoria
*Ce. cacaofunesta*	C 1593	Mating-type switching [38]	GCA_002776505	30.5	Molano et al. 2018 [52]
*Ce. colombiana*	CBS 121792	Mating-type switching [38]	GCA_026419805	31.1	Wingfield et al. 2022 [53]
*Ce. eucalypticola*	CMW 9998	Mating-type switching [38]	GCA_001513815	30.7	Wingfield et al. 2015 [54]
*Ce. fimbriata*	CBS 114723	Mating-type switching [38]	GCA_000389695	30.0	Wilken et al. 2013 [55]
	CMW 15049	Mating-type switching [38]	GCA_012652265 ^1^	28.1	University of Pretoria
	LPF 1912	Mating-type switching [38]	GCA_009914735 ^1^	31.6	Santos et al. 2020 [56]
	UHS-CF5	Mating-type switching [38]	GCA_023525945 ^1^	33.0	University of Horticultural Sciences
	NC 236	Mating-type switching [38]	GCA_017160715 ^1^	27.8	Michigan State University
	AS236	Mating-type switching [38]	GCA_033035045 ^1^	31.7	Stahr et al. 2024 [57]
*Ce. harringtonii*	CMW 14789	Mating-type switching [38]	GCA_002018265	26.1	Wingfield et al. 2016 [48]
*Ce. lukuohia*	CBS 142792	Mating-type switching [38]	GCA_023509845	30.7	Iowa State University
*Ce. manginecans*	CMW 17570	Mating-type switching [38]	GCA_000712455 ^1^	31.7	van Der Nest et al. 2014 [51]
	CMW 22563	Mating-type switching [38]	GCA_006681795 ^1^	31.6	University of Pretoria
	CMW 46461	Mating-type switching [38]	GCA_006408425	31.9	Fourie et al. 2019 [58]
	C 4621	Mating-type switching [38]	GCA_026122545 ^1^	31.3	Iowa State University
*Ce. platani*	CFO	Mating-type switching [38]	GCA_000978885	29.1	University of Neuchatel
*Ce. smalleyi*	CMW 14800	Mating-type switching [38]	GCA_003449175	25.8	Wingfield et al. 2018 [46]
*Chalaropsis populi*	CMW 26388	Heterothallic [43]	GCA_017591655	23.9	van Der Nest et al. 2021 [59]
*Ch. thielavioides*	JCM 1933	Heterothallic [43]	GCA_001599435	23.3	RIKEN Center for Life Science Technologies
*Davidsoniella australis*	CMW 2333	Heterothallic [43]	GCA_009806335	34.1	Forestry and Agricultural Biotechnology Institute
*D. eucalypti*	CMW 3254	Heterothallic [43]	GCA_004009845	41.4	Forestry and Agricultural Biotechnology Institute
*D. neocaledoniae*	CMW 26392	Heterothallic [43]	GCA_009806295	33.5	Forestry and Agricultural Biotechnology Institute
*D. virescens*	CMW 17339	Mating-type switching [38]	GCA_001513805	33.4	Wingfield et al. 2015 [54]
*Endoconidiophora laricicola*	CBS 100207	Mating-type switching [38]	GCA_001640655	32.7	Wingfield et al. 2016 [49]
*E. polonica*	CBS 100205	Mating-type switching [38]	GCA_001856765	32.5	Wingfield et al. 2016 [49]
*Huntiella abstrusa*	CMW 21092	Heterothallic [60]	GCA_025685495 ^2^	29.4	Wingfield et al. 2022 [61]
*H. bhutanensis*	CMW 8217	Heterothallic [62]	GCA_002018275 ^2^	26.7	Wingfield et al. 2016 [48]
*H. decipiens*	CMW 30855	Heterothallic [40]	GCA_003032515 ^2^	26.6	Wingfield et al. 2017 [63]
*H. fecunda*	CMW 49301	Unisexual [32]	GCA_027744815 ^2^	25.0	Wilson et al. 2023 [40]
*H. moniliformis*	CBS 118127	Unisexual [9]	GCA_000712465 ^2^	25.4	van Der Nest et al. 2014 [51]
*H. omanensis*	CMW 11056	Heterothallic [9]	GCA_000833645 ^2^	31.1	van Der Nest et al. 2014 [64]
*H. savannae*	CBS 121151	Heterothallic [32]	GCA_001483325 ^2^	28.5	van Der Nest et al. 2015 [65]
*H. tyalla*	CMW 28920	Unisexual [40]	GCA_027744755 ^2^	24.9	Wilson et al. 2023 [40]
*Knoxdaviesia capensis*	CMW 40890	N/A to study	GCA_001510575 ^3^	35.5	Aylward et al. 2016 [66]
*K. proteae*	CMW 40885	N/A to study	GCA_001510565 ^3^	35.5	Aylward et al. 2016 [67]
*Meredithiella fracta*	CBS 142645	Mating-type switching [43]	GCA_023677585	27.0	Duong et al. 2021 [68]
*M. norrisii*	CBS 139737	Mating-type switching [43]	JAXARZ000000000	28.7	Rakoma 2024 [43]
*Phialophoropsis hubbardii*	CBS 408.68	Primary homothallic [43]	JAXCEM000000000	26.8	Rakoma 2024 [43]
*Thielaviopsis cerberus*	CMW 36653	Mating-type switching [36]	GCA_016859225	28.6	Krämer et al. 2021 [36]
*Th. ethacetica*	JCM 6961	Heterothallic [37]	GCA_001599055	29.5	RIKEN Center for Life Science Technologies
*Th. euricoi*	JCM 6020	Heterothallic [37]	GCA_001599615	29.6	RIKEN Center for Life Science Technologies
*Th. musarum*	CMW 1546	Heterothallic [37]	GCA_001513885	28.4	Wingfield et al. 2015 [54]
*Th. punctulata*	CMW 1032	Heterothallic [37]	GCA_002925815	27.8	Wilken et al. 2018 [37]
	CR-DP1	Heterothallic [37]	GCA_000968615 ^1^	28.1	University of Illinois
	DSM 102798	Heterothallic [37]	GCA_036034705 ^1^	28.2	United Arab Emirates University
*Toshionella taiwanensis*	CBS 141494	Mating-type switching [43]	JAXCEL000000000	32.5	Rakoma 2024 [43]
*Wolfgangiella franznegeri*	CBS 144149	Mating-type switching [43]	JAXCEK000000000	27.2	Rakoma 2024 [43]

^1^ Genome assembly was not used to make the phylogenetic tree

^2^ Genome assemblies only used for the purpose of making the multigene phylogeny and to extend loci from previously published data

^3^ These genome assemblies were used as an outgroup (family *Gondwanamycetaceae*) in this study

The sexual reproductive strategy of most species was assigned based on published literature (Table 1). For species where no sexual state has been defined, mating strategies were assigned based on available *MAT1* gene content and structure [43]. Heterothallism was assigned to species where individuals with either the *MAT1-1* or *MAT1-2* idiomorph were identified [2]. Primary homothallic species were characterised by having both *MAT1-1-1* and *MAT1-2-1* genes at the same locus, with no evidence of repeat elements that could indicate *MAT1* locus instability [2]. Species with mating-type switching had a *MAT1* locus structure similar to primary homothallism, but were distinguished by two repeat elements that flank the *MAT1-2* genes, and in some cases disrupt the *MAT1-1-1* coding sequence [38].

### Construction of a phylogenomic tree for the *Ceratocystidaceae* and outgroup species

A robust and updated phylogenetic tree was generated to provide the backbone for later analysis and to visualize the distribution of mating strategies across the family. For this purpose, a multigene phylogeny was constructed from genomic sequences using a series of custom Bash scripts (code can be accessed on GitHub at https://github.com/FrancesLane/SordariomycetesBUSCOphylogeny). The phylogeny was based on a set of BUSCO genes present in 45 genome assemblies that included the 43 *Ceratocystidaceae* species (one representative isolate for each species; Table 1), and those of *K. capensis* and *K. proteae* as outgroups. BUSCO genes were predicted (v. 2.0.1) [69] in all genome assemblies using the sordariomycetes_odb10 lineage BUSCO library that was filtered to include only the first isoform per reference gene. BUSCO genes predicted in all 45 genome assemblies were concatenated and the nucleotide sequences were aligned using MAFFT (v. 7.407) [70], after which the alignments were trimmed with TrimAl (v. 1.4.rev22) [71]. The trimmed alignments were then concatenated using FASconCAT-G (v. 1.05.1) [72] with MrBAYES parameters. The phylogeny was constructed by IQTree2 (v. 2.2.5) [73, 74] using the MFP model finder and a bootstrap of 1 000 replicates, and visualised with iTOL [75].

### Identification of genes involved in the pheromone response pathway

Previously characterised proteins of the a- and α-pheromone proteins [40, 76] and their cognate receptors (Suppl. Table 2), as well as proteins that play a role in pheromone processing and the signal transduction pathway (Suppl. Table 3) were used to identify contigs that contained these genes in the target genomes. The proteins from each set were used as query sequences in tBLASTn searches of the custom genome assembly database but excluding the genomes of the *Huntiella* species that were analysed previously [40]. These searches were conducted with CLC Main Workbench (v. 22.0.2), and the “mask low complexity regions” setting was disabled for the pheromone protein searches.

Regions of interest identified by the BLAST results, as well as the regions flanking it, were submitted to AUGUSTUS (*Fusarium graminearum* gene model) [77] for *de novo* gene prediction. In cases where the contig was small (< 15 kb for the receptors and < 30 kb for the pheromones), the entire contig was submitted for annotation. When a putative open reading frame (ORF) for a pheromone gene was identified by BLAST, but could not be predicted by AUGUSTUS, a similarity-based gene prediction was attempted using FGENESH+ (with the generic *Fusarium* gene models and a close relative’s pheromone protein) [78]. To confirm the identity of the predicted genes, each one was translated *in silico* and used as a query in a BLASTp search against the NCBI nr protein database. To identify the genomic location of the pheromone and pheromone-receptor genes, either one (for the receptors) or three (for the pheromones) genes to either side of the target gene was also annotated.

Where no contigs containing a putative pheromone gene were identified using the initial tBLASTn searches, two additional approaches were used. Firstly, the newly identified *Ceratocystidaceae* pheromone proteins were used as queries in new tBLASTn searches of the genomes. Alternatively, a microsynteny-based approach was used that entailed tBLASTn searches of the genome assemblies using protein sequences of genes flanking the pheromone gene in close relatives, including flanking genes described in *Huntiella* [40]. Any contigs identified were used for gene prediction with AUGUSTUS or FGENESH+. If no ORF for the a-pheromone gene was predicted by either tool, the region where the gene would be expected was translated into all six reading frames. CaaX/CpaX motifs positioned immediately before a stop codon were identified and any codons for methionine within 300 bp (corresponding to 100 amino acids) upstream of the stop codon were considered as a putative start codon for possible a-pheromone genes.

**Fig. 1:**
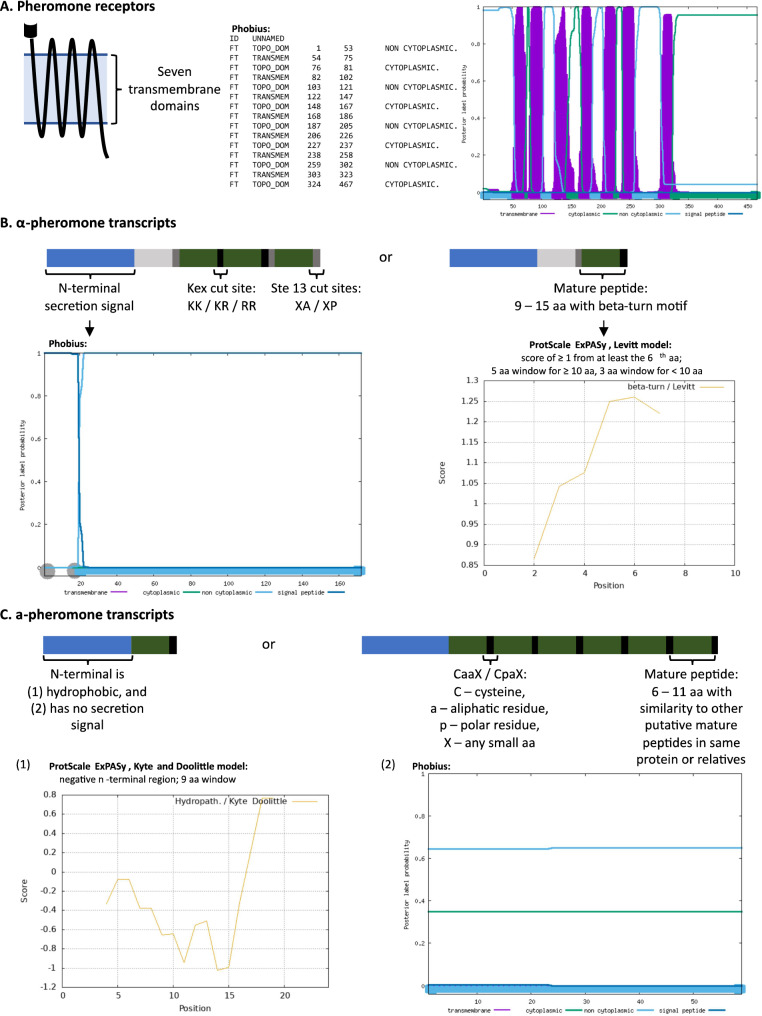
The characteristics and methods used to confirm the identity of a putative pheromone or pheromone receptor and infer putatively functionality. **A** Simple illustration of the pheromone receptor protein containing seven transmembrane domains (left), confirmed by Phobius output (right). **B** Diagram representing that structure of the α-pheromone protein with single and multiple mature peptides. It has an N-terminal secretion signal represented by the dark blue line in the Phobius output, mature peptide units with beta-turn motifs (ProtScale ExPASy output illustrating this using the Levitt model) that are flanked by KEX and STE13 cut sites. **C** Diagram representing the characteristics of the a-pheromone protein encoding one or more mature peptides. The N-terminus is (1) hydrophobic, indicated by the Kyte and Doolittle model, and has no secretion signal (2) based on Phobius output. All a-pheromone proteins must have a terminal CaaX/CpaX site, and where multiple mature peptides were predicted, they were all followed by a CaaX/CpaX site as well

The predicted proteins had to fulfil some criteria to be considered putatively functional pheromones or pheromone receptors (Fig. 1). These were evaluated using a combination of ProtScale ExPASy [79], Phobius [80] and manual annotation (Fig. 1; Suppl. File 1). To be considered functional, both pheromone receptors had to have the characteristic seven transmembrane domains. An α-pheromone gene was considered as correct if the putative protein had a N-terminal secretion signal and mature-peptide sequences flanked by KEX and/or STE13 cleavage sites. Possible mature α-pheromone peptides were identified by taking between nine and fifteen amino acids upstream of cleavage sites and screening these for the presence of a predicted beta-turn motif (Fig. 1). The putative a-pheromone protein had to have an N-terminus that was hydrophobic and lacked a signal peptide, and a C-terminal with a CaaX/CpaX motif (Fig. 1). Six to eleven amino acids immediately preceding any CaaX/CpaX motifs together with the cysteine residue (the C notation in the CaaX/CpaX motif) were designated as a putative mature pheromone. The presence of any similar repeats of this sequence either within the same protein, or within other putative *Ceratocystidaceae* a-pheromone proteins, were also taken as support of the prediction. When conserved domains, cleavage sites, or other indicators of putative function were absent, the coding sequence was manually checked for gaps (regions represented by strings of Ns). When available, raw reads were mapped to these regions to assess base-calling fidelity.

### Comparative analyses of pheromones, pheromone-receptors, their loci, and mating strategies

The pheromone receptors, pheromones and mature pheromone peptides were compared based on their amino acid sequence conservation, overall structure, and genomic location to identify if there were any unique characteristics that could be correlated to mating strategy. The amino acid conservation between both the pheromone-receptor proteins and the mature pheromone peptides elucidated in this study was determined for the members of a single genus. To do this, these sequences were aligned using the built-in alignment tool from CLC Main Workbench, and the pairwise percent identity was calculated using default settings. A *Ceratocystidaceae*-wide alignment of the mature pheromone peptides was created using the online version of MAFFT (v. 7) [81, 82] and visualised in CLC Main Workbench. Microsynteny in the loci containing the predicted pheromone and pheromone-receptor genes was visualised in a pair-wise comparison using Clinker [83]. This output was plotted onto the multigene tree representing the phylogenetic relationship and sexual strategies of the *Ceratocystidaceae* and related taxa. Various characteristics of the pheromones and pheromone receptors were compared across species sharing the same mating strategy. These included (i) the position of each locus, (ii) putative functionality of the encoded receptors (based on the presence of seven transmembrane domains), (iii) the predicted protein and mature pheromone-peptide size, (iv) number of mature peptides encoded by a single gene, and (v) gene copy variations.

## Results

### Construction of a phylogenomic tree for the *Ceratocystidaceae* and outgroup species

**Fig. 2:**
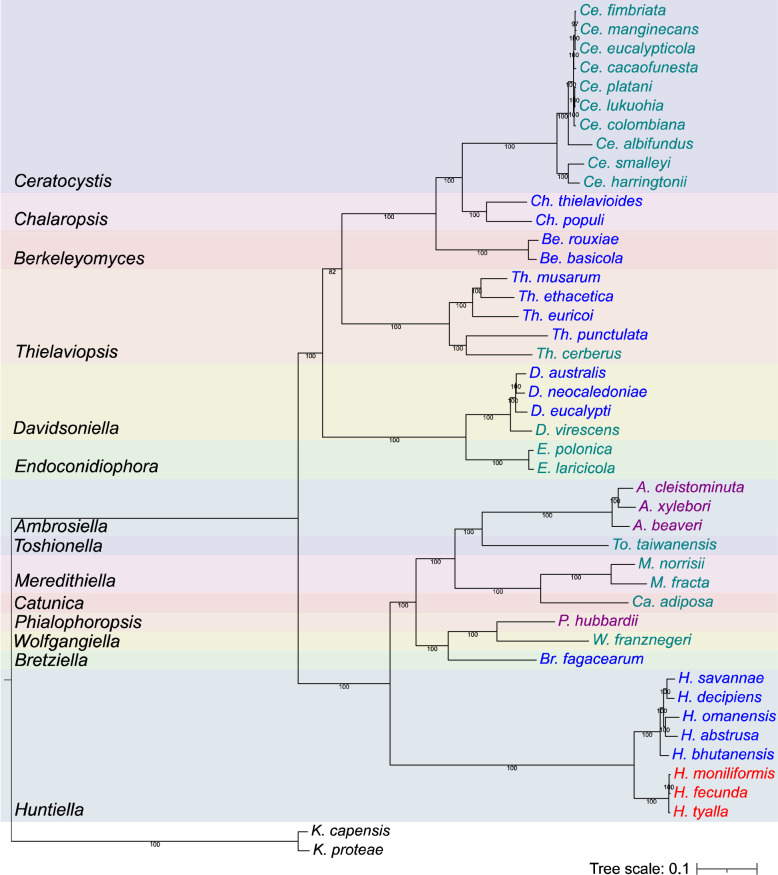
A phylogenetic tree of the *Ceratocystidaceae* family. The phylogeny was generated using 1 284 *Sordariomycetes* BUSCO genes that were predicted in all genome assemblies and produced using the MFP model finder with a bootstrap of 1 000 replicates. *Knoxdaviesia capensis* and *K. proteae* were used as outgroup species. Sexual strategies are indicated by the colour of the species name: heterothallic = blue, primary homothallic = purple, mating-type switching = green, unisexual = red

The phylogeny produced for the *Ceratocystidaceae* provided the backbone against which the pheromones, their receptors and their respective loci could be compared (Fig. 2). From the *Sordariomycetes* BUSCO database, 1 284 complete genes were present in all the *Ceratocystidaceae* and *Gondwanamycetaceae* genomes included in the phylogeny (Table 1). The multigene phylogeny was well supported by 100% bootstrap values across all branches except for the node separating *Thielaviopsis* from the clade containing *Berkeleyomyces*,* Ceratocystis* and *Chalaropsis* (82% support), and between *Ce. fimbriata* and *Ce. manginecans* (97% support; Fig. 2).

### Identification of genes involved in the pheromone response pathway

#### The pheromone-receptor genes and loci

**Fig. 3:**
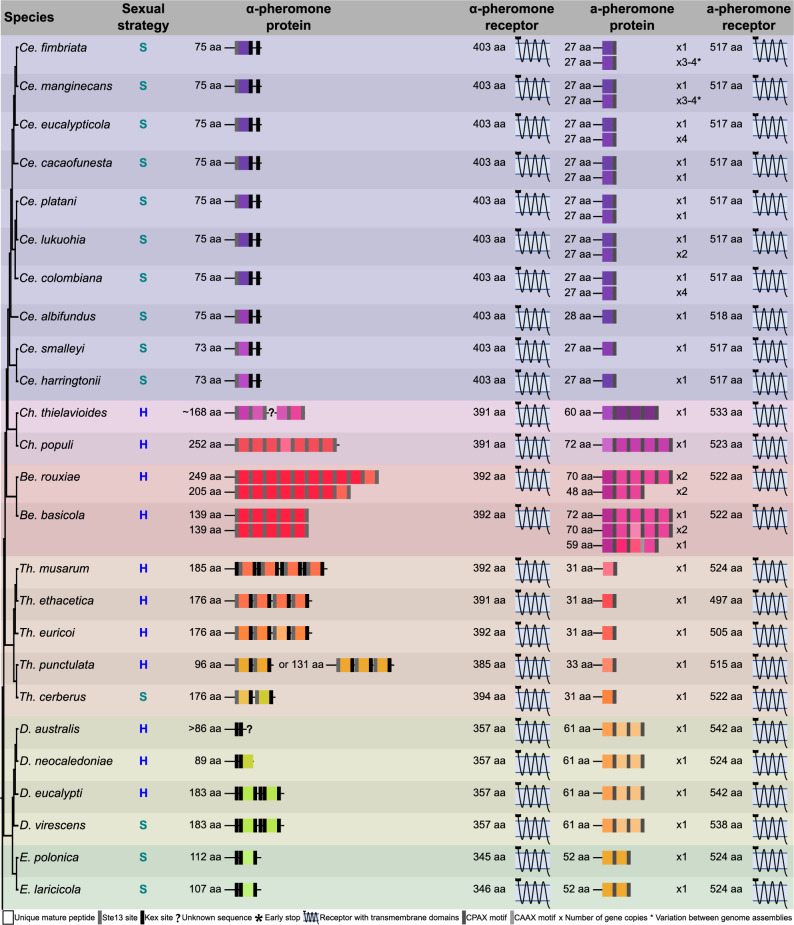

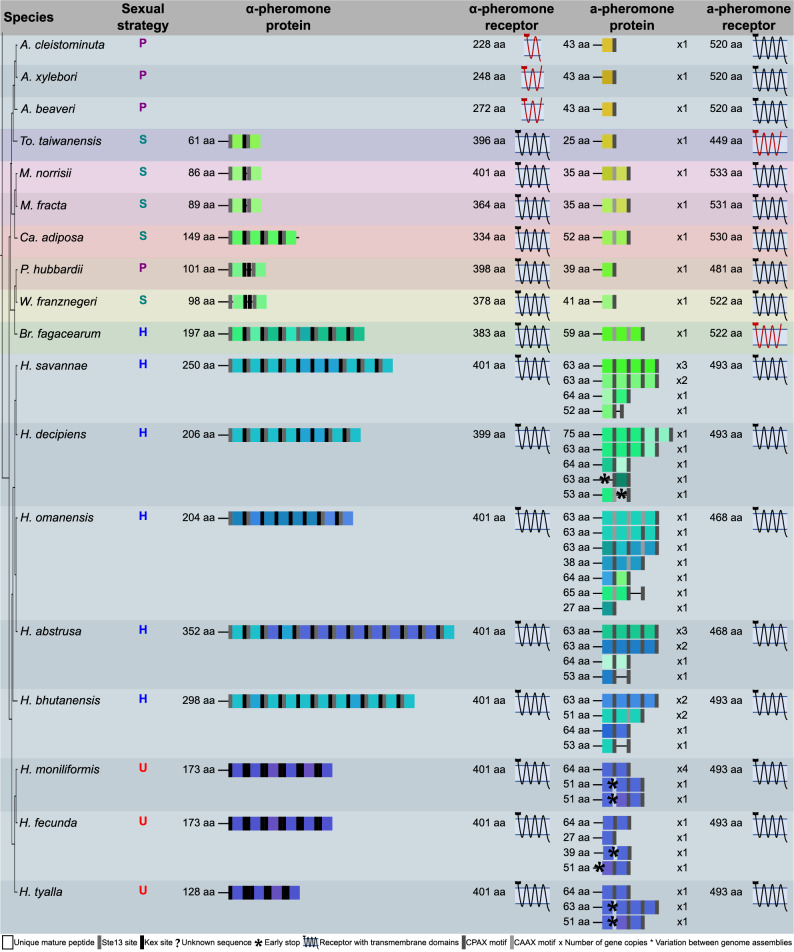
A visual summary of the pheromones and pheromone-receptor proteins of the *Ceratocystidaceae* (not drawn to scale). The sexual strategies are denoted as H for heterothallic, P for primary homothallic, S for mating-type switching, and U for unisexual. Mature pheromone peptides are indicated by coloured squares where each colour represents a unique amino acid sequence, and the grey to black rectangles indicate the type of cleavage site/motif that likely mediates the release of the mature peptides (see bottom legend for colour coding of the different cleavage sites/motifs). An asterisk indicates an early stop codon, and unknown sequence due to the end of a contig is indicated with a question mark. The sizes of the unprocessed pheromone proteins are indicated on the left of the pheromone diagrams and the number of copies of genes encoding the proteins are indicated on the right (e.g., x2). A diagrammatic representation of the receptors with their predicted transmembrane domains is provided with the size of the protein give on the left. The cell membrane is indicated by the blue bar with the extracellular region being above the bar and the cytoplasmic region being below. Putatively functional receptors are drawn in black while non-functional receptors are red

Both the a- and α-pheromone-receptor genes were identified in all the genome assemblies used in this study (Fig. 3), and these were deemed putatively functional in all except five species. The a-pheromone-receptor gene encoded only six of the seven transmembrane domains in *Bretziella fagacearum* and *Toshionella taiwanensis*. Although the complete ORF was similar in length to the a-pheromone receptors of relatives from the same phylogenetic clades that had seven transmembrane domains, the last domain (TM7) could not be predicted as it had several key mutations in this region (Suppl. Figure 1). The α-pheromone-receptor genes in all the *Ambrosiella* genomes encoded only three or four of the expected seven transmembrane domains, making these proteins significantly shorter than that of *To. taiwanensis*. This was due to multiple deletions in regions that corresponded to the seven transmembrane domains of *To. taiwanensis* (Suppl. Figure 1), although these deletions were generally unique to each *Ambrosiella* species. The loss of these transmembrane domains in the a- or α-pheromone receptors resulted in the C-terminus being extracellular rather than cytoplasmic, as is the case in functional pheromone receptors, in all cases apart from *A. cleistominuta*. In the latter species, a cytoplasmic C-terminus was still predicted as only three domains were present.

Comparisons of the amino acid sequence of the a- and α-pheromone receptors revealed a high level of conservation at the genus level for both proteins (> 79% sequence identity; Suppl. Figures 2 and 3), with only two clear exceptions. The α-pheromone receptor of the *Ambrosiella* species showed lower conservation (28% to 52%), likely due to the unique deletions discussed earlier (Suppl. Figure 1). *Thielaviopsis* also exhibited lower conservation for both receptors, with sequence similarity as low as 56% for the a-pheromone receptor, although the sequence variation did not change the structure of the transmembrane domains. Where multiple genomes were available for a species (Table 1), small levels of intraspecific variation were found in both pheromone-receptor proteins, with a minimum sequence similarity of 97% between the a-pheromone receptors of *Th. punctulata* (Suppl. Figure 3).

**Fig. 4:**
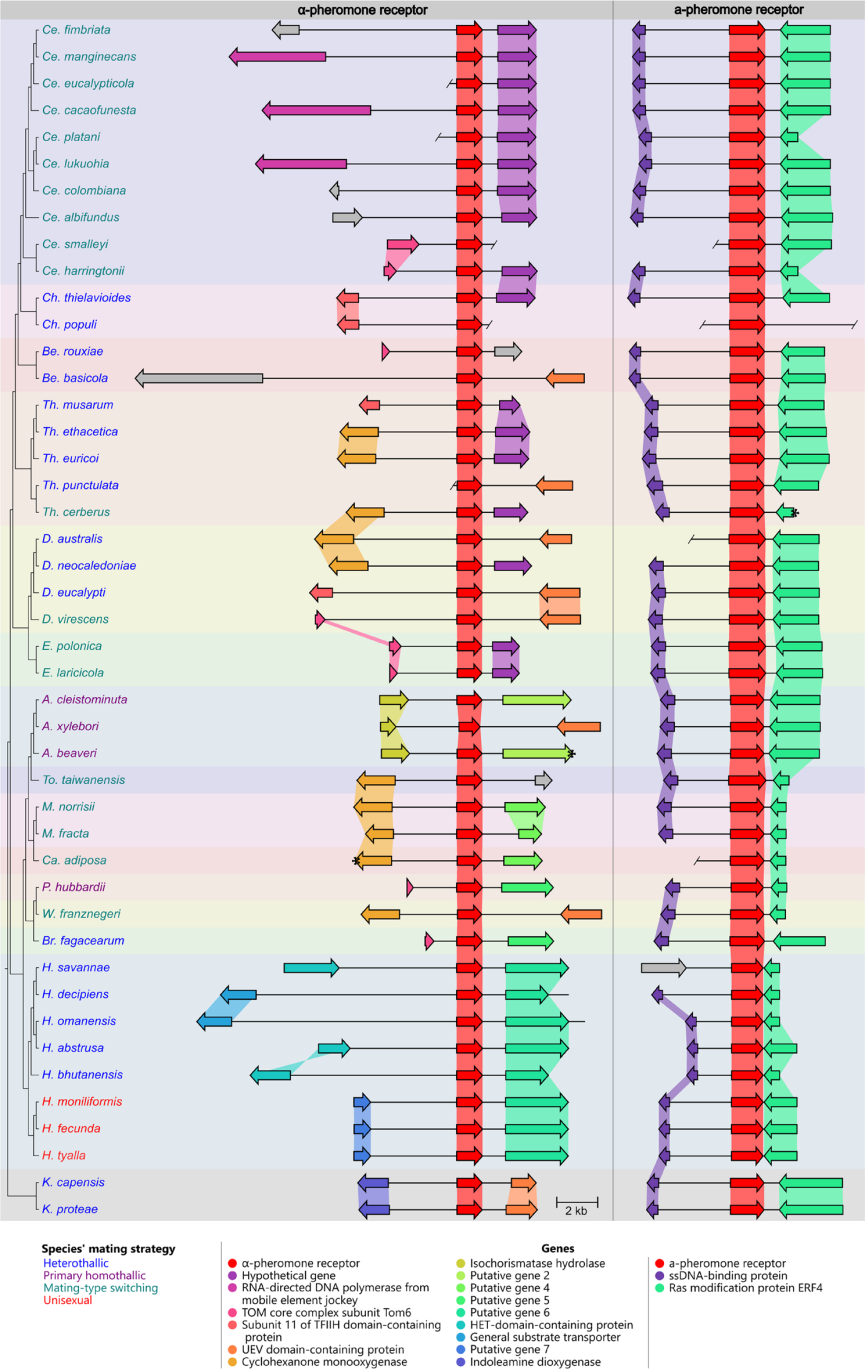
A pairwise comparison of the a-pheromone receptor and α-pheromone receptor loci. The region compared included the pheromone-receptor gene and two flanking genes where possible. Ends of contigs are indicated with a slash or an asterisk when the end of the of a contig was within a gene. “Hypothetical” genes were named as such if a BLAST result was to a protein listed as “hypothetical” on NCBI, while “putative genes” were genes that were predicted but had no valid BLAST result. These genes were numbered based on their order of appearance in the figure. Genes that did not have homology to any other genes in the dataset are indicated as grey arrows

The flanking genes proved useful in establishing the genomic position for the two pheromone-receptor genes. The same two genes flanked the a-pheromone-receptor gene in almost all species, indicating a family-level conservation in the genomic position of this gene (Fig. 4; Additional File 1). An exception was in *H. savannae* that had a unique gene in the upstream region between the a-pheromone receptor and the upstream gene found in all other species (data not shown). The genes flanking the α-pheromone-receptor gene were significantly more variable (Fig. 4; Additional File 2), making identification of a single locus conserved across the family impossible. Rather, synteny of the α-pheromone-receptor loci appeared to be linked to taxonomic relationship, where species from a single genus would often share flanking genes.

#### The α-pheromone genes and loci

A putative α-pheromone gene was identified in most genome assemblies with the only exception being the three *Ambrosiella* species (Fig. 3). In most cases the predicted transcripts exhibited many characteristics of other α-pheromone proteins that would indicate correct processing and transportation of the mature pheromone peptides. This included multiple mature α-pheromone peptides of nine to twelve amino acids in length, flanked by KEX and/or STE13 cleavage sites. Each mature peptide had a conserved central glycine residue, except for *Meredithiella* species that had a central tryptophan residue. In addition, all predicted peptides had a beta-turn. This indicates that these pheromone genes likely encode functional mature pheromone peptides that may be important to the sexual cycle of these fungi.

There was substantial variation between the number and sequence of the mature α-pheromone peptides among the different genera, but this variation was less apparent among species of the same genus (Fig. 3). Across the family, a single α-pheromone gene could encode between one and thirteen mature pheromone peptides (Suppl. File 2), and contained either only KEX or STE13 sites, or a combination of both. Intraspecific variability was observed among the *Th. punctulata* isolates (Fig. 3) where two isolates had an α-pheromone gene encoding three mature peptides while the third isolate encoded only two. Where multiple mature peptides were encoded by the same gene, in many cases the repeats were similar but not always identical (Fig. 3). The most extreme case was in *Br. fagacearum* that had five unique mature peptides. The mature peptides were also often identical or similar between members of the same genus, but could still have an identity as low as 55% in some genera (Suppl. Figure 4). Comparisons across the entire *Ceratocystidaceae* revealed a high level of variation, with as many as 38 unique mature α-pheromone peptides (Suppl. Figure 5). For the most part, the mature peptides were only conserved within a single genus, with one exception being a shared mature peptide between *Phialophoropsis* and *Wolfgangiella*.

**Fig. 5:**
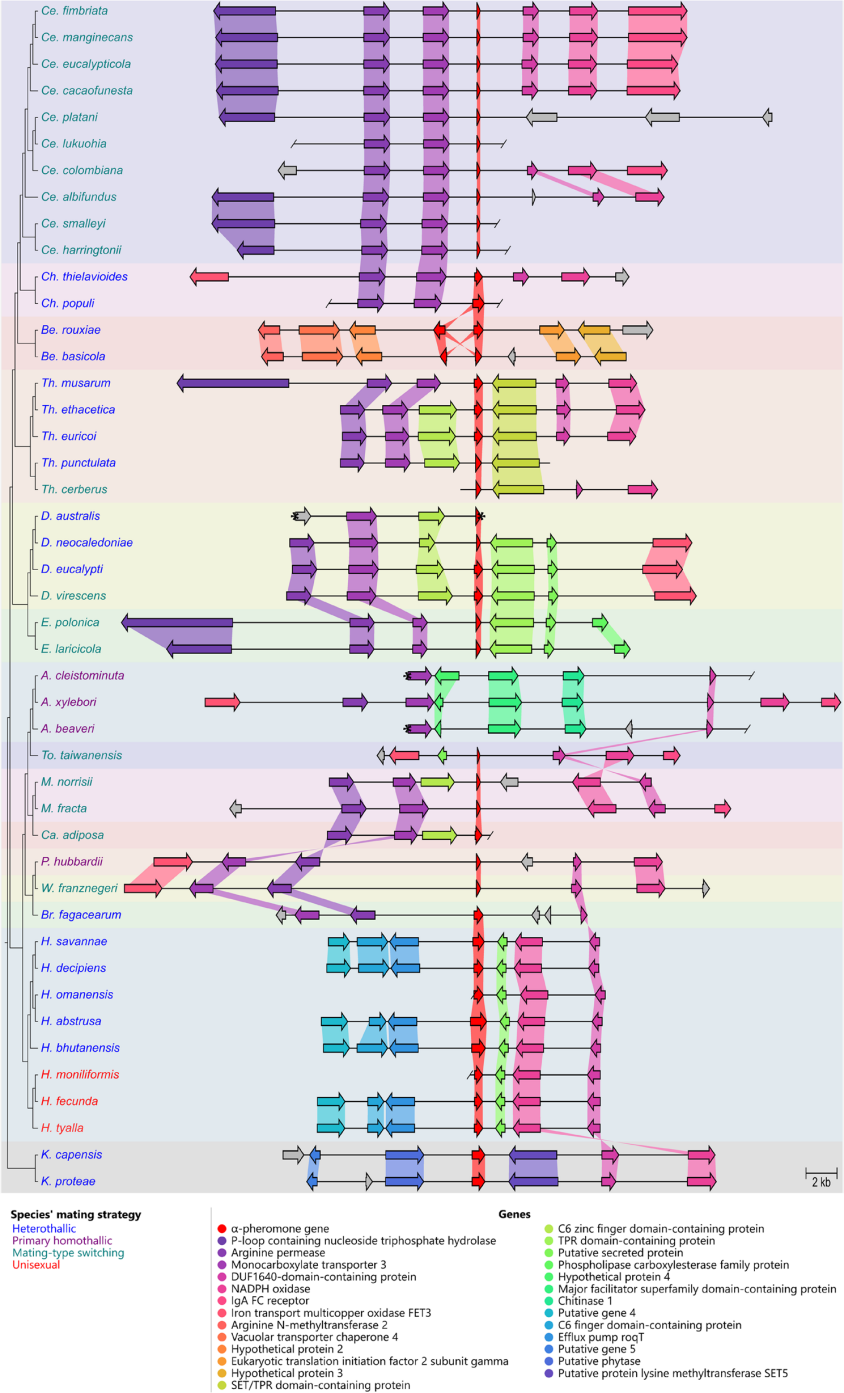
A pairwise comparison of the α-pheromone locus. The α-pheromone gene with three genes flanking either side where possible is shown. Ends of contigs are indicated with a slash or an asterisk when the end of the of a contig was within a gene. “Hypothetical” genes were named as such if a BLAST result was to a protein listed as “hypothetical” on NCBI, while “putative gene” were genes that were predicted but had no valid BLAST result. These genes were numbered based on their order of appearance in the figure. Genes that did not have homology to any other genes in the dataset are indicated as grey arrows

A family-conserved locus was apparent for the α-pheromone gene (Fig. 5; Additional File 3), although distinct variation was present in two genera. This was evident from the presence of a conserved set of genes upstream of the α-pheromone gene in all species except *Huntiella* and *Toshionella taiwanensis*. These species had unique genes upstream of the α-pheromone gene but had genes downstream that were homologous to those of close relatives. The α-pheromone gene could not be identified in any of the three *Ambrosiella* genomes, even after attempts to manually identify the gene in the family-conserved locus. In contrast, both *Berkeleyomyces* species contained two inverted copies of the α-pheromone gene at a single locus that is distinct from the rest of the family. The two copies encoded identical α-pheromone proteins in *Be. basicola* while the two α-pheromone proteins encoded by *Be. rouxiae* differed by 44 amino acids, representing two mature α-pheromone peptides (Fig. 3). At the family-conserved α-pheromone locus, both *Berkeleyomyces* had genes that are associated with transposable elements and retroviruses in place of where the α-pheromone gene would be expected (Suppl. File 3).

#### The a-pheromone genes and loci

Either a single copy or multiple copies of the a-pheromone gene were identified in all the *Ceratocystidaceae* genome assemblies (Fig. 3). All identified pheromones encoded a hydrophobic N-terminus with no signal peptide and a C-terminal CaaX or CpaX site, indicating that the proteins will be processed and transported correctly to produce functional mature a-pheromone peptides. Where an a-pheromone gene encoded for multiple mature peptides, each peptide was followed by its own CaaX/CpaX motif in addition to the terminal motif (Fig. 3). An interesting variation to the standard CpaX motif was noted in *Catunica adiposa* and *Meredithiella* species where the polar residue (p) was a glutamine rather than the more common polar residues (asparagine, serine or threonine) reported previously for this motif [84].

Similar to the other pheromone, the mature a-pheromone peptides exhibited a large amount of variation in sequence and copy number (Fig. 3), although this diversity was lower at the genus level. A single a-pheromone gene could encode between one and five mature peptides of between seven and ten amino acids in length. Where multiple a-pheromone genes were present in a genome, the number of mature peptides was not always the same. The mature a-pheromone peptides were often similar in sequence but not identical, resulting in 50 different mature a-pheromone peptides that could be identified across the *Ceratocystidaceae* (Suppl. Figure 5). Most sequence variation between the members of a single genus was found in *Berkeleyomyces* and *Huntiella* (minimum percentage identity of 50% and 44% respectively; Suppl. Figure 6), although most genera showed conservation of 70% or more. Notably, at least one mature peptide variant could be identified as present in all a-pheromone genes across the members of a genus (Fig. 3), although no mature a-pheromone peptide sequences were shared across genus boundaries.

**Fig. 6:**
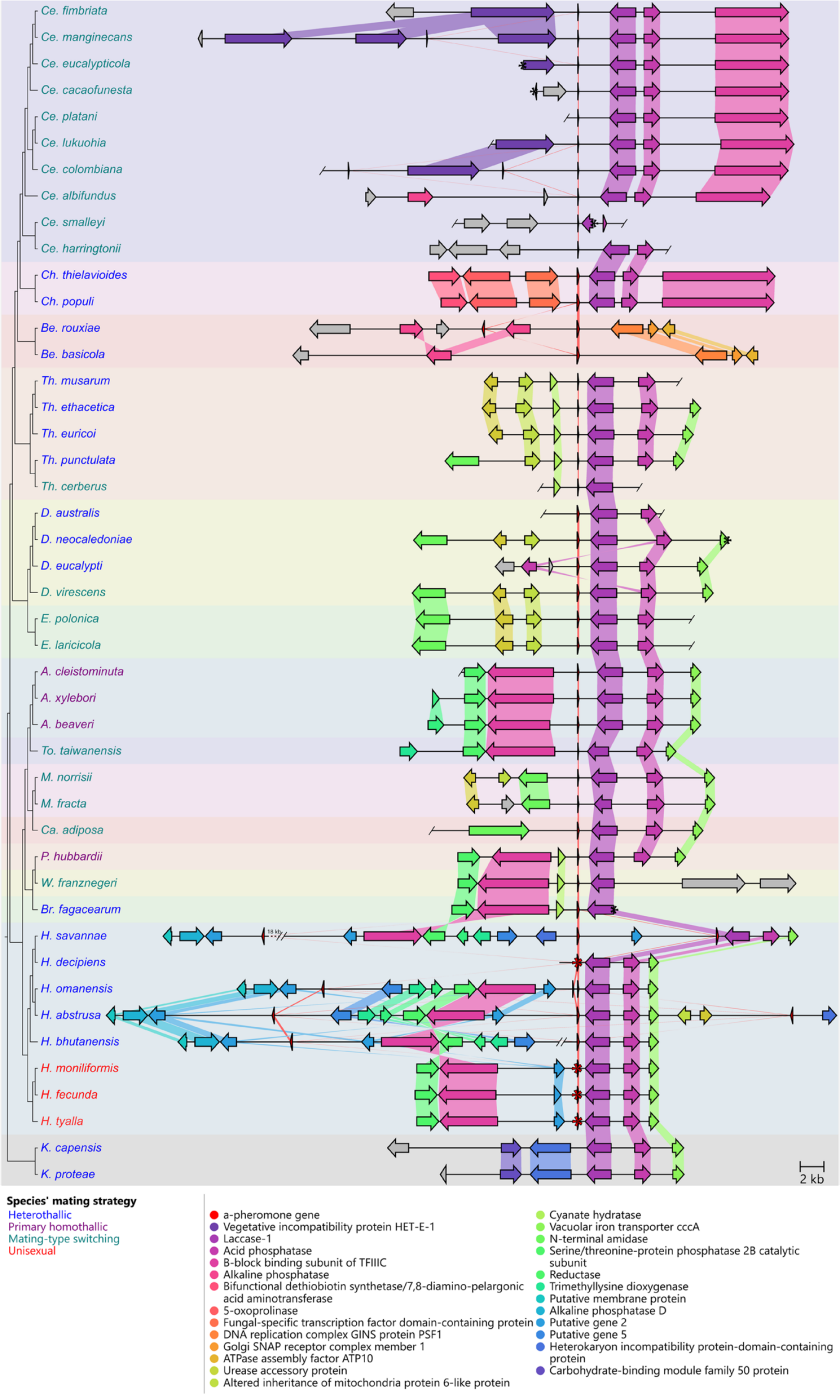
A pairwise comparison of the a-pheromone locus. The a-pheromone gene with three genes flanking either side where possible is shown. Ends of contigs are indicated with a slash and a black asterisk when the end of the of a contig was within a gene, or the gene was truncated. Red asterisks indicated a-pheromone genes with early stop codons. In some cases, contigs were joined (indicated by two adjacent ends of contigs) based on close relatives, and in one case (*H. savannae*), the contigs were joined but approximately 18 kb of sequence was omitted (indicated with a dashed line) for visual purposes. “Putative gene” were genes that were predicted but had no valid BLAST result, and were numbered based on their order of appearance in the figure. Genes that did not have homology to any other genes in the dataset are indicated as grey arrows

One locus containing between one and three copies of the a-pheromone gene was conserved in almost all *Ceratocystidaceae* species. This locus was defined by a laccase-1 gene that was present in the region downstream of the a-pheromone gene (Fig. 6; Additional File 4), with more variability in the gene content upstream of the locus. Three species showed more variability in terms of the genes flanking the a-pheromone gene. The a-pheromone gene in *Ce. smalleyi* had unique upstream genes, and only a truncated remnant of the laccase-1 gene (5’ truncation) was detected downstream. The genes flanking the a-pheromone locus in both *Berkeleyomyces* species were unique apart from one upstream gene that was also detected in the locus of *Ce. albifundus*. In *H. decipiens*, *H. fecunda*, *H. moniliformis* and *H. tyalla*, a copy of the a-pheromone gene with an early stop codon was identified at this locus, but these species have putatively functional a-pheromone gene copies elsewhere in the genome [40] (Fig. 3).

Similar to that reported for species of *Huntiella* [40], multiple a-pheromone loci were identified in nine more *Ceratocystidaceae* species considered in this study. This included the two *Berkeleyomyces* and multiple *Ceratocystis* species (Suppl. Figure 7; Additional File 5). In *Be. basicola*, an additional two loci were located on separate contigs, each with a single copy of the a-pheromone gene, while *Be. rouxiae* had only one additional locus. This locus was homologous to one of the *Be. basicola* loci, but with two copies of the a-pheromone about 30.5 kb apart. Between one and five additional a-pheromone genes were present and mostly shared between the *Ceratocystis* species *Ce. cacaofunesta*, *Ce. colombiana*, *Ce. eucalypticola*, *Ce. fimbriata*, *Ce. lukuohia*, *Ce. manginecans* and *Ce. platani* (Suppl. File 4). Intraspecific variation in the a-pheromone loci were also observed in *Ce. fimbriata* and *Ce. manginecans* which had six and four genome assemblies available respectively (Suppl. Figure 8), although no intraspecies variation was found in other species for which multiple (between two and seven; Table 1) genome assemblies were available. Most intraspecific variation was limited to the identity of the flanking genes, but in *Ce. manginecans* isolate UHS-CF5 a unique tandem duplication of the a-pheromone gene was identified (Suppl. Figure 8, locus 3).

#### Additional pheromone signalling pathway genes

To further support the presence of a putatively functional pheromone/receptor system, genes known to be important for the functioning of the pheromone/receptor signalling pathway and mature pheromone peptide processing were identified. A BLASTp search of all the genome sequences followed by gene prediction identified these homologs in all 54 *Ceratocystidaceae* genome assemblies (Suppl. Table 3). In cases where the complete coding gene sequence could not be predicted due to its location at the end of a contig, BLAST queries using the partial protein product was sufficient to confirm gene presence.

### Comparative analyses of pheromones, pheromone-receptors, their loci, and mating strategies

Comparisons of the genomic locations, predicted protein structure and presence/absence of functional mating pheromones and pheromone-receptors among heterothallic, primary homothallic, and mating-type switching *Ceratocystidaceae* did not reveal clear patterns specific to each sexual strategy, even within the same genus (Fig. 3, Suppl. Table 4). For instance, the two pheromone genes in *D. virescens*, a species using unidirectional mating-type switching [85], were near-identical in size, mature peptide number, and sequence to those of its heterothallic sister species, *D. australis*, *D. eucalypti* and *D. neocaledoniae* [86]. Similarly, *Th. cerberus*, which also switches mating types [36], had similar mature a- and α-peptide sequences to the heterothallic *Thielaviopsis* relatives [29, 37], although *Th. cerberus* did encode fewer mature α-pheromone peptides. In contrast, of the primary homothallic *Ceratocystidaceae*, only *Phialophoropsis hubbardii* [43] had functional pheromone/receptor pathway genes. Heterothallic individuals also varied in the sequence and mature peptide number of their pheromone genes, much like their homothallic relatives.

There was no link to mating strategy where pheromone/receptor genes were missing or non-functional in a species. While the missing and non-functional genes observed in *Ambrosiella* species may be tolerated by their primary homothallic lifestyle [35], the loss of their a-pheromones and cognate receptors appears to be linked to lineage-specific events. Uncharacteristically for a heterothallic species, *Br. fagacearum* had a non-functional a-pheromone receptor, whereas all other heterothallic species had functional pheromone/receptor genes. *Wolfgangiella franznegeri* was the only mating-type switching species to lack a functional a-pheromone receptor. Sexual reproduction has been reported in this species [87], suggesting that the sexual cycle is not affected by this loss. The unusual placement of the pheromone genes in *Berkeleyomyces* did not appear linked to its heterothallic lifestyle, although it may play a role in the lack of an observable sexual state in mating studies [33].

All genes necessary for a functional pheromone/receptor pathway were present in at least one representative *Ceratocystidaceae* species for each of the mating strategies. The presence of a typical pheromone/receptor response pathway, including all processing genes, together with observable sexual reproduction in many of these species [23, 35, 87] suggests that all mating strategies analysed in this study likely draw on this pathway to produce sexual progeny. Variability observed in the structure of the proteins themselves, especially in the case of the pheromones, is most apparent when comparisons are made between genera rather than mating strategy (Fig. 3). For example, although the same mating strategy was shared by *D. virescens* and *Th. cerberus*, the a-pheromone proteins differed in both sequence and structure. In contrast, these proteins were structurally identical to that of relatives within the same genus, despite them differing in mating strategies. This appears to point to changes in the pheromone/receptor system following speciation patterns rather than driven by sexual reproductive strategy.

## Discussion

This study considered whether variation in the architecture of the pheromone/receptor loci could be linked to mating strategy in the *Ceratocystidaceae*. This question arose from a previous study on *Huntiella*, a genus in the same family of fungi, that found structural differences in the α-pheromone protein between heterothallic and unisexual species, suggesting mating-strategy specific modifications [40]. However, in our study, identification and comparisons of the pheromone and cognate receptor genes in 43 species of *Ceratocystidaceae* representing four distinct mating strategies, showed no clear association between these factors. Such an association would have been expected considering the role of the pheromone/receptor response pathway in fungal sexual reproduction [2, 3, 11]. Although the considerable variation observed in the pheromone proteins and their gene locations across the entire *Ceratocystidaceae* appeared congruent with speciation, there may be a connection to the respective mating strategies and sexual behaviours that we were not able to identify.

Of the genes in the pheromone/receptor response pathway, the pheromones showed the most variation between genera. This included variation in the number of genes present in a genome, as well as differences in the amino acid sequence and number of mature peptides produced from a gene. The rapid divergence of genes involved in gamete recognition of animals, fungi and plants [76, 88, 89] could explain this variability as it is important in establishing fertilisation barriers that ultimately drive speciation [90]. However, many *Ceratocystidaceae* species had identical or near identical mature pheromone peptide sequences, mirroring findings in *Fusarium* [76]. In both yeasts and filamentous ascomycetes, different species in a single genus can produce identical pheromones while maintaining reproductive barriers [11, 91, 92]. Therefore, post-mating compatibility may be more important than premating barriers to avoid hybridisation [93]. Laboratory-derived and natural hybrids have been reported in *Ceratocystis* and *Endoconidiophora* [94–96], and the highly similar pheromone peptides may have allowed interspecific mate recognition. In the *Ceratocystidaceae*, pheromone divergence is more likely a product of speciation rather than its cause.

The patterns of pheromone variation observed suggest that different evolutionary forces contribute to their diversification in the *Ceratocystidaceae*. In *Fusarium*, diversity in pheromone genes has been attributed to a combination of positive selection and relaxed selective constraints [76], processes that can drive rapid sequence change. Although the repetitive nature of pheromone genes and the presence of multiple copies could promote concerted evolution, where repeats are homogenised across the genome [97], the divergent mature peptide repeats and gene copies reported in *Fusarium* led the authors to propose a birth-and-death model of evolution [76]. This model, in which continual duplication and loss events generate genetic variability within and between species [98], aligns with the mature peptide number and sequence diversity seen in the *Ceratocystidaceae*. For example, *Th. punctulata* isolates differed in the number of mature α-pheromone peptides encoded, and the a-pheromone gene of *H. omanensis* that encoded seven unique mature peptides, consistent with birth-and-death dynamics shaping pheromone evolution in this family.

The observed variability in the positioning of the pheromone genes emphasises the dynamic nature of this locus. This was most apparent in *Berkeleyomyces*, where the pheromone genes were present at loci unique to this genus, and the α-pheromone gene was duplicated. As both *Berkeleyomyces* species share this locus, the gene movement and duplication are likely ancestral to the genus rather than a recent event. Variability was also seen in the positions of the a-pheromone genes when multiple gene copies were present. Although one gene was mostly present at a common locus, a varied number of additional copies was found within additional loci. Intraspecific variation was also observed in *Ce. fimbriata* and *Ce. manginecans*, including one *Ce. fimbriata* isolate with two gene copies at a locus where all other isolates had only one, suggesting a recent duplication event. Pheromone genes are well-known for gene duplications, deletions, and mobility [91], and in the *Saccharomycetaceae* 19 unique a-pheromone gene locations were reported across 23 species [99]. Some genomic rearrangements could also result from translocation events driven by retroviruses and transposable elements [100]. Similar translocations have been found in other fungi [101], including the retrotransposon-mediated movement of *MAT1-2-1* to a separate chromosome in *Neurospora sublineolata* [102].

Synteny analysis of the pheromone and pheromone-receptor loci revealed strong positional conservation for the a-pheromone receptor, whereas the alternative receptor and both pheromone genes showed no consistent synteny. This pattern suggests that the flanking regions of these genes may have undergone dynamic rearrangements over evolutionary time. Similar rearrangements around the pheromone and receptor (P/R) locus have been reported in the basidiomycete *Cryptococcus amylolentus* and related *Cryptococcus* species [103]. In addition, a whole-genome comparison of three *Aspergillus* species showed that approximately 22% of their assemblies lacked long-range synteny, with micro-rearrangements occurring even within conserved syntenic blocks [104]. Such structural changes are often associated with repetitive sequences, particularly in pericentric and subtelomeric regions [105]. Although the proximity of these loci to centromeres or telomeres, and the potential effects of local rearrangements, were beyond the scope of this study, these factors may represent promising directions for future research.

Some of the pheromone genes identified in the *Ceratocystidaceae* differed structurally from those previously described in other filamentous ascomycetes. In the genera *Ceratocystis*, *Davidsoniella* and *Endoconidiophora*, the α-pheromone gene could encode a single mature peptide and not multiple copies as reported in all other filamentous ascomycetes studied [11, 18, 106]. The mature α-pheromone peptides of *Meredithiella* contained a central tryptophan residue within the core motif, which is different to the more common glycine amino acid associated with this pheromone [11, 107]. Some a-pheromone proteins contained only a single cleavage site (either STE13 or KEX), instead of both sites that are usually needed to release the mature peptides [18]. These findings not only provide a broader definition of what constitutes pheromone genes, but also expands our understanding of how these could be processed in filamentous fungi. This could also inform future attempts aimed at identifying these genes in other fungi, a task that has proven difficult in the past [76, 92, 108, 109].

The apparent lack of a direct link between mating strategy and the pheromone/receptor genes could possibly be explained by expression differences, although this was not investigated in the present study. For example, heterothallic species generally have mating-type-specific expression of the pheromones [12–14] and, in some cases, the pheromone-receptor genes [15, 110], while all genes are ubiquitously expressed in primary homothallic fungi [20, 111]. In the *Ceratocystidaceae* specifically, the heterothallic *H. omanensis* has alternative splicing of a receptor and pheromone expression, both in a mating-type specific fashion [39]. In contrast, the unisexual fungus *H. moniliformis* displays simultaneous expression of both pheromones [39]. The now annotated pheromone and pheromone-receptor genes together with existing transcriptomic data of *Ceratocystidaceae* species [39, 40, 45, 112] could be used in future studies to explore how expression differences in the pheromone/receptor system might influences the mating behaviour of fungi with varied mating strategies.

The absence of the α-pheromone gene and the degeneration of its cognate receptor in *Ambrosiella* suggest that this component of the pheromone system is no longer required in these species. Although *A. cleistominuta* retains a predicted cytoplasmic C-terminus, all *Ambrosiella* α-pheromone receptors lack key transmembrane domains and extracellular loop regions known to mediate pheromone binding and signalling [113, 114], strongly indicating loss of function. Similar loss of pheromone-receptor pairs has been observed in other ascomycetes while maintaining a sexual cycle [19–21, 115], suggesting a relaxed selection on these genes once they become dispensable. Sexual reproduction observed in *A. cleistominuta* and *A. xylebori* [35, 87] supports the view that the α-pheromone pathway has been bypassed in this lineage. The species-specific patterns of receptor gene erosion imply independent loss events, consistent with a shift in selective pressures rather than a single ancestral mutation [76, 116]. Given the specialised beetle-associated lifecycle of *Ambrosiella* [117], relaxed selection on the α-pheromone signalling pathway may reflect ecological adaptation, although this has not occurred in other ambrosial *Ceratocystidaceae*, indicating a lineage-specific trajectory.

Future studies should explore additional roles of the pheromone-receptor pathway in the life cycle of *Ceratocystidaceae* species. In other ascomycetes, these pathways significantly influence biological processes like mating and fertility [19–22]. In *Fusarium* species, the pheromone-receptor system has been repurposed for non-mating functions, such as host detection through root exudates [118, 119]. Given that some *Ceratocystidaceae* are soil-borne pathogens [120–122], investigating its role in host infection is promising. Established transformation systems in various members of this group [123–128] could facilitate functional characterization of pheromones and receptors. Such studies would deepen our understanding of the biology of the *Ceratocystidaceae* and reveal novel roles for the pheromone-response pathway in closely related species having different mating strategies.

The pheromone and receptor genes were accurately identified within the limitations of the available data. Functionality was inferred based on conserved domains and processing motifs, but should be validated through functional studies. Significant intraspecific variation was detected in some taxa, but multiple genome assemblies for other species would be needed to determine if this is a common trait for these genes. Expression of pheromone and receptor genes have previously been demonstrated in *Huntiella* species using RNA sequencing [39, 40]. They should also be performed on other *Ceratocystidaceae* species using publicly (SRA database; https://www.ncbi.nlm.nih.gov/sra) and targeted gene expression datasets. The conservation of synteny among these genes suggests that the gene models presented in this study are correct and would simplify expression analysis. Future work aiming to investigate expression may require high-depth, short-read sequencing to reliably capture the short a-pheromone transcripts.

## Supplementary Information

Supplementary Material 1.

Supplementary Material 2.

Supplementary Material 3.

Supplementary Material 4.

Supplementary Material 5.

Supplementary Material 6. Treatment of predicted pheromone receptors lacking seven transmembrane domains.

Supplementary Material 7. Explanation for ambiguity in mature α-pheromone peptide numbers.

Supplementary Material 8. Genes found in the family-conserved α-pheromone locus of Berkeleyomyces species and variation in one Be. rouxiae genome assembly.

Supplementary Material 9. Details on additional a-pheromone loci identified in Ceratocystis species.

Supplementary Material 10. Genome assembly QUAST and BUSCO scores.

Supplementary Material 11. Supplementary Table 2: GenBank accession numbers for the pheromone-receptor proteins used as query sequences to identify homologs in the Ceratocystidaceae genome assemblies. Supplementary Table 3: Proteins involved in the pheromone response pathway that were used to identify homologs in genome assemblies investigated. Supplementary Table 4: Consolidation of findings to identify any correlation between mating strategy and pheromone-receptor system in Ceratocystidaceae species.

Supplementary Material 12. Supplementary Figure 1: Alignments of the pheromone-receptor proteins from species lacking all seven transmembrane domains with pheromone-receptor proteins from species within the same clade where all seven domains were predicted. (A) The a-pheromone receptor of Br. fagacearum compared with P. hubbardiid and W. franznegeri. (B) The a-pheromone receptor of To. taiwanensis aligned to Ambrosiella proteins. (C) The α-pheromone receptor of Ambrosiella aligned to that of To. taiwanensis. Predicted extracellular regions are indicated by yellow rectangles, cytoplasmic regions by purple and transmembrane domains by blue. Black blocks highlight regions where transmembrane domains are absent or adjacent domains may have merged into one. Supplementary Figure 2: A pairwise comparison showing the percentage identity of the α-pheromone receptor proteins between members of a genus. Supplementary Figure 3: A pairwise comparison showing the percentage identity of the a-pheromone receptor proteins between members of a genus. Supplementary Figure 4: A pairwise comparison showing the percentage identity of the mature α-pheromone peptides between members of a genus. Supplementary Figure 5: An alignment of the putative a- and α-pheromone mature peptides. Species are numbered according to their order from top to bottom on the phylogeny (Fig. 1). Supplementary Figure 6: A pairwise comparison showing the percentage identity of the mature a-pheromone peptides between members of a genus.

## Data Availability

All scripts used to create the multigene phylogeny using *Sordariomycete* BUSCO genes can be found at https://github.com/FrancesLane/SordariomycetesBUSCOphylogeny. All genome assemblies used are available on the NCBI Genome database (see Table 1 for accession numbers). All pheromone and pheromone-receptor loci identified and annotated in this study are available in the supplementary material and its additional files.

## References

[1] Billiard S, Lopez-Villavicencio M, Devier B, Hood ME, Fairhead C, Giraud T. Having sex, yes, but with whom? Inferences from fungi on the evolution of anisogamy and mating types. Biol Rev. 2011;86:421–42. 21489122 10.1111/j.1469-185X.2010.00153.x

[2] Wilson AM, Wilken PM, Wingfield MJ, Wingfield BD. Genetic networks that govern sexual reproduction in the pezizomycotina. Microbiol Mol Biol Rev. 2021;85:e0002021. 34585983 10.1128/MMBR.00020-21 PMC8485983

[3] Dyer PS, Inderbitzin P, Debuchy R. Mating-type structure, function, regulation and evolution in the pezizomycotina. In: Wendland J, editor. Growth, differentiation and sexuality. Cham, Switzerland: Springer; 2016. pp. 315–85.

[4] Stanton BC, Hull CM. Mating-type locus control of cell identity. In: Heitman J, Kronstad JW, Taylor JW, Casselton LA, editors. Sex in fungi: molecular determination and evolutionary implications. Washington, USA: ASM; 2007. pp. 59–73.

[5] Metzenberg RL, Glass NL. Mating type and mating strategies in *Neurospora*. Bioessays. 1990;12:53–9. 2140508 10.1002/bies.950120202

[6] Ni M, Feretzaki M, Sun S, Wang X, Heitman J. Sex in fungi. Annu Rev Genet. 2011;45:405–30. 21942368 10.1146/annurev-genet-110410-132536 PMC3310392

[7] Wilson AM, Wilken PM, van der Nest MA, Steenkamp ET, Wingfield MJ, Wingfield BD. Homothallism: an umbrella term for describing diverse sexual behaviours. IMA Fungus. 2015;6:207–14. 26203424 10.5598/imafungus.2015.06.01.13 PMC4500084

[8] Lin X, Heitman J. Mechanisms of homothallism in fungi and transitions between heterothallism and homothallism. In: Heitman J, Kronstad JW, Taylor JW, Casselton LA, editors. Sex in fungi: molecular determination and evolutionary implications. Washington, USA: ASM; 2007. pp. 35–57.

[9] Wilson AM, Godlonton T, van der Nest MA, Wilken PM, Wingfield MJ, Wingfield BD. Unisexual reproduction in *Huntiella moniliformis*. Fungal Genet Biol. 2015;80:1–9. 25910452 10.1016/j.fgb.2015.04.008

[10] Bennett RJ, Turgeon BG. Fungal sex: the *Ascomycota*. Microbiol Spectr. 2016;4:10–128. 10.1128/microbiolspec.FUNK-0005-2016 27763253

[11] Pöggeler S. 5 Function and evolution of pheromones and pheromone receptors in filamentous ascomycetes. In: Pöggeler S, Wöstemeyer J, editors. Evolution of Fungi and Fungal-Like Organisms. The Mycota. Springer: Berlin, Heidelberg; 2011;14:73–96.

[12] Bobrowicz P, Pawlak R, Correa A, Bell-Pedersen D, Ebbole DJ. The *Neurospora crassa* pheromone precursor genes are regulated by the mating type locus and the circadian clock. Mol Microbiol. 2002;45:795–804. 12139624 10.1046/j.1365-2958.2002.03052.x

[13] Zhang L, Baasiri RA, Van Alfen NK. Viral repression of fungal pheromone precursor gene expression. Mol Cell Biol. 1998;18:953–9. 9447992 10.1128/mcb.18.2.953 PMC108807

[14] Shen WC, Bobrowicz P, Ebbole DJ. Isolation of pheromone precursor genes of *Magnaporthe grisea*. Fungal Genet Biol. 1999;27:253–63. 10441451 10.1006/fgbi.1999.1151

[15] Kim H, Wright SJ, Park G, Ouyang S, Krystofova S, Borkovich KA. Roles for receptors, pheromones, G proteins, and mating type genes during sexual reproduction in *Neurospora crassa*. Genetics. 2012;190:1389–404. 22298702 10.1534/genetics.111.136358 PMC3316651

[16] Seibel C, Tisch D, Kubicek CP, Schmoll M. The role of pheromone receptors for communication and mating in *Hypocrea jecorina* (*Trichoderma reesei*). Fungal Genet Biol. 2012;49:814–24. 22884620 10.1016/j.fgb.2012.07.004 PMC3462998

[17] Xue C, Hsueh YP, Heitman J. Magnificent seven: roles of G protein-coupled receptors in extracellular sensing in fungi. FEMS Microbiol Rev. 2008;32:1010–32. 18811658 10.1111/j.1574-6976.2008.00131.x PMC2998294

[18] Jones SK Jr., Bennett RJ. Fungal mating pheromones: choreographing the dating game. Fungal Genet Biol. 2011;48:668–76. 21496492 10.1016/j.fgb.2011.04.001 PMC3100450

[19] Kim HK, Lee T, Yun SH. A putative pheromone signaling pathway is dispensable for self-fertility in the homothallic ascomycete *Gibberella zeae*. Fungal Genet Biol. 2008;45:1188–96. 18567512 10.1016/j.fgb.2008.05.008

[20] Lee J, Leslie JF, Bowden RL. Expression and function of sex pheromones and receptors in the homothallic ascomycete *Gibberella Zeae*. Eukaryot Cell. 2008;7:1211–21. 18503004 10.1128/EC.00272-07 PMC2446672

[21] Mayrhofer S, Weber JM, Poggeler S. Pheromones and pheromone receptors are required for proper sexual development in the homothallic ascomycete *Sordaria macrospora*. Genetics. 2006;172:1521–33. 16387884 10.1534/genetics.105.047381 PMC1456310

[22] Seo JA, Han KH, Yu JH. The *gprA* and *gprB* genes encode putative G protein-coupled receptors required for self-fertilization in *Aspergillus nidulans*. Mol Microbiol. 2004;53:1611–23. 15341643 10.1111/j.1365-2958.2004.04232.x

[23] de Beer ZW, Duong TA, Barnes I, Wingfield BD, Wingfield MJ. Redefining *Ceratocystis* and allied genera. Stud Mycol. 2014;79:187–219. 25492989 10.1016/j.simyco.2014.10.001 PMC4255530

[24] Marín Montoya M, Wingfield MJ. A review of *Ceratocystis sensu stricto* with special reference to the species complexes *C. coerulescens* and *C. fimbriata*. Rev Fac Nac Agron Medellin. 2006;59:3045–375.

[25] Wingfield BD, van Wyk M, Roos H, Wingfield MJ. *Ceratocystis*: emerging evidence for discrete generic boundaries. In: Seifert KA, de Beer ZW, Wingfield MJ, editors. The ophiostomatoid fungi: expanding frontiers. Volume 90. Utrecht, The Netherlands: CBS-KNAW Fungal Biodiversity Centre; 2013. pp. 57–64.

[26] van Wyk M, Al Adawi AO, Khan IA, Deadman ML, Al Jahwari AA, Wingfield BD, et al. *Ceratocystis manginecans* sp. nov., causal agent of a destructive mango wilt disease in Oman and Pakistan. Fungal Divers. 2007;27 I:213–30.

[27] Nel WJ, Duong TA, Wingfield BD, Wingfield MJ, de Beer ZW. A new genus and species for the globally important, multihost root pathogen *Thielaviopsis basicola*. Plant Pathol. 2018;67:871–82.

[28] Redfern DB, Stoakley JT, Steele H, Minter DW. Dieback and death of larch caused by *Ceratocystis laricicola* sp. nov. following attack by *Ips cembrae*. Plant Pathol. 2007;36:467–80.

[29] Mbenoun M, de Beer ZW, Wingfield MJ, Wingfield BD, Roux J. Reconsidering species boundaries in the *Ceratocystis paradoxa* complex, including a new species from oil palm and cacao in Cameroon. Mycologia. 2014;106:757–84. 24987122 10.3852/13-298

[30] Roux J, van Wyk M, Hatting H, Wingfield MJ. *Ceratocystis* species infecting stem wounds on *Eucalyptus grandis* in South Africa. Plant Pathol. 2004;53:414–21.

[31] Baker CJ, Harrington TC, Krauss U, Alfenas AC. Genetic variability and host specialization in the Latin American clade of *Ceratocystis fimbriata*. Phytopathology. 2003;93:1274–84. 18944327 10.1094/PHYTO.2003.93.10.1274

[32] Liu F, Li G, Roux J, Barnes I, Wilson AM, Wingfield MJ, et al. Nine novel species of *Huntiella* from Southern China with three distinct mating strategies and variable levels of pathogenicity. Mycologia. 2018;110:1145–71. 30431409 10.1080/00275514.2018.1515450

[33] Nel WJ, Duong TA, Wingfield MJ, Wingfield BD, Hammerbacher A, de Beer ZW. Heterothallism revealed in the root rot fungi *Berkeleyomyces basicola* and *B. rouxiae*. Fungal Biol. 2018;122:1031–40. 30342619 10.1016/j.funbio.2018.08.006

[34] Wilken PM, Steenkamp ET, Wingfield MJ, de Beer ZW, Wingfield BD. DNA loss at the *Ceratocystis fimbriata* mating locus results in self-sterility. PLoS One. 2014;9:e92180. 24651494 10.1371/journal.pone.0092180 PMC3961304

[35] Mayers CG, Harrington TC, Ranger CM. First report of a sexual state in an ambrosia fungus: *Ambrosiella cleistominuta* sp. nov. associated with the ambrosia beetle *Anisandrus maiche*. Botany. 2017;95:503–12.

[36] Krämer D, Lane FA, Steenkamp ET, Wingfield BD, Wilken PM. Unidirectional mating-type switching confers self-fertility to *Thielaviopsis cerberus*, the only homothallic species in the genus. Fungal Biol. 2021;125:427–34. 34024590 10.1016/j.funbio.2020.12.007

[37] Wilken PM, Steenkamp ET, van der Nest MA, Wingfield MJ, de Beer ZW, Wingfield BD. Unexpected placement of the *MAT1-1-2* gene in the *MAT1-2* idiomorph of *Thielaviopsis*. Fungal Genet Biol. 2018;113:32–41. 29409964 10.1016/j.fgb.2018.01.007

[38] Wilken PM, Lane FA, Steenkamp ET, Wingfield MJ, Wingfield BD. Unidirectional mating-type switching is underpinned by a conserved *MAT1* locus architecture. Fungal Genet Biol. 2024;170:103859. 38114017 10.1016/j.fgb.2023.103859

[39] Wilson AM, van der Nest MA, Wilken PM, Wingfield MJ, Wingfield BD. Pheromone expression reveals putative mechanism of unisexuality in a saprobic ascomycete fungus. PLoS One. 2018;13:e0192517. 29505565 10.1371/journal.pone.0192517 PMC5837088

[40] Wilson AM, Wingfield MJ, Wingfield BD. Structure and number of mating pheromone genes is closely linked to sexual reproductive strategy in *Huntiella*. BMC Genomics. 2023;24:261. 37179314 10.1186/s12864-023-09355-9 PMC10182648

[41] Mikheenko A, Prjibelski A, Saveliev V, Antipov D, Gurevich A. Versatile genome assembly evaluation with QUAST-LG. Bioinformatics. 2018;34:i142–50. 29949969 10.1093/bioinformatics/bty266 PMC6022658

[42] Manni M, Berkeley MR, Seppey M, Simao FA, Zdobnov EM. BUSCO update: novel and streamlined workflows along with broader and deeper phylogenetic coverage for scoring of eukaryotic, prokaryotic, and viral genomes. Mol Biol Evol. 2021;38:4647–54. 34320186 10.1093/molbev/msab199 PMC8476166

[43] Rakoma JR. Structure of the mating-type locus in ambrosial and asexual *Ceratocystidaceae* species. Dissertation. University of Pretoria; 2024.

[44] Wilken PM, Aylward J, Chand R, Grewe F, Lane FA, Sinha S et al. IMA Genome - F13: Draft genome sequences of *Ambrosiella cleistominuta*, *Cercospora brassicicola*, *C. citrullina*, *Physcia stellaris*, and *Teratosphaeria pseudoeucalypti*. IMA Fungus. 2020;11:19. 10.1186/s43008-020-00039-7 PMC7513301 33014691

[45] Vanderpool D, Bracewell RR, McCutcheon JP. Know your farmer: ancient origins and multiple independent domestications of ambrosia beetle fungal cultivars. Mol Ecol. 2018;27:2077–94. 29087025 10.1111/mec.14394

[46] Wingfield BD, Bills GF, Dong Y, Huang W, Nel WJ, Swalarsk-Parry BS, et al. IMA Genome-F 9: draft genome sequence of annulohypoxylon stygium, *Aspergillus mulundensis*, *Berkeleyomyces basicola *(syn. *Thielaviopsis basicola*), *C**eratocystis smalleyi*, two *C**ercospora beticola *strains, *Coleophoma cylindrospora*, *Fusarium fracticaudum*,* Phialophora* cf. *hyalina*, and *Morchella septimelata*. IMA Fungus. 2018;9:199–223. 30018880 10.5598/imafungus.2018.09.01.13 PMC6048567

[47] Dumigan CR, Maddock S, Bray-Stone D, Deyholos MK. Hybrid genome assembly of *Berkeleyomyces rouxiae*, an emerging *Cannabis* fungal pathogen causing black root rot in an aeroponic facility. Plant Dis. 2023;107:2679–86. 36774565 10.1094/PDIS-11-22-2690-RE

[48] Wingfield BD, Duong TA, Hammerbacher A, van der Nest MA, Wilson A, Chang R, et al. IMA Genome-F 7: draft genome sequences for *Ceratocystis fagacearum*, *C. harringtonii*, *Grosmannia penicillata*, and *Huntiella bhutanensis*. IMA Fungus. 2016;7:317–23. 27990338 10.5598/imafungus.2016.07.02.11 PMC5159602

[49] Wingfield BD, Ambler JM, Coetzee MP, de Beer ZW, Duong TA, Joubert F, et al. IMA Genome-F 6: draft genome sequences of * A**rmillaria fuscipes*, *C**eratocystiopsis minuta*, *C**eratocystis adiposa*, *E**ndoconidiophora laricicola*, *E. polonica *and *P**enicillium freii *DAOMC 242723. IMA Fungus. 2016;7:217–27. 27433447 10.5598/imafungus.2016.07.01.11 PMC4941685

[50] van der Nest MA, Steenkamp ET, Roodt D, Soal NC, Palmer M, Chan WY, et al. Genomic analysis of the aggressive tree pathogen *Ceratocystis albifundus*. Fungal Biol. 2019;123:351–63. 31053324 10.1016/j.funbio.2019.02.002

[51] van Der Nest MA, Bihon W, De Vos L, Naidoo K, Roodt D, Rubagotti E, et al. IMA Genome-F 2: draft genome sequences of *Diplodia sapinea*, *Ceratocystis manginecans*, and *Ceratocystis moniliformis*. IMA Fungus. 2014;5:135–40. 25083413 10.5598/imafungus.2014.05.01.13 PMC4107891

[52] Molano EPL, Cabrera OG, Jose J, do Nascimento LC, Carazzolle MF, Teixeira P, et al. *Ceratocystis **c**acaofunesta* genome analysis reveals a large expansion of extracellular phosphatidylinositol-specific phospholipase-C genes (PI-PLC). BMC Genomics. 2018;19:58. 29343217 10.1186/s12864-018-4440-4 PMC5773145

[53] Wingfield BD, Berger DK, Coetzee MPA, Duong TA, Martin A, Pham NQ, et al. IMA genome–F17: draft genome sequences of an *Armillaria* species from Zimbabwe, *Ceratocystis colombiana*, *Elsinoë necatrix*, *Rosellinia necatrix*, two genomes of *Sclerotinia minor*, short–read genome assemblies and annotations of four *Pyrenophora Teres* isolates from barley grass, and a long-read genome assembly of *Cercospora zeina*. IMA Fungus. 2022;13:19. 36411457 10.1186/s43008-022-00104-3 PMC9677705

[54] Wingfield BD, Barnes I, de Beer ZW, De Vos L, Duong TA, Kanzi AM et al. IMA Genome-F 5: Draft genome sequences of *Ceratocystis eucalypticola*, *Chrysoporthe cubensis*, *C. deuterocubensis*, *Davidsoniella virescens*, *Fusarium temperatum*, *Graphilbum fragrans*, *Penicillium nordicum*, and *Thielaviopsis musarum*. IMA Fungus. 2015;6:493–506. 10.5598/imafungus.2015.06.02.13 PMC4681265 26734552

[55] Wilken PM, Steenkamp ET, Wingfield MJ, de Beer ZW, Wingfield BD. IMA genome-F 1: *Ceratocystis fimbriata*: draft nuclear genome sequence for the plant pathogen, *Ceratocystis fimbriata*. IMA Fungus. 2013;4:357–8. 24563841 10.5598/imafungus.2013.04.02.14 PMC3905947

[56] Santos SA, Vidigal PMP, Thrimawithana A, Betancourth BML, Guimaraes LMS, Templeton MD, et al. Comparative genomic and transcriptomic analyses reveal different pathogenicity-related genes among three eucalyptus fungal pathogens. Fungal Genet Biol. 2020;137:103332. 31926322 10.1016/j.fgb.2019.103332

[57] Stahr M, Parada-Rojas CH, Childs K, Alfenas R, Fernandes FM, Avila K, et al. Long-read sequencing genome assembly of *Ceratocystis fimbriata* enables development of molecular diagnostics for sweetpotato black rot. Phytopathology. 2024. . 10.1094/PHYTO-09-23-0341-R 38264989

[58] Fourie A, van der Nest MA, de Vos L, Wingfield MJ, Wingfield BD, Barnes I. QTL mapping of mycelial growth and aggressiveness to distinct hosts in *Ceratocystis* pathogens. Fungal Genet Biol. 2019;131:103242. 31212023 10.1016/j.fgb.2019.103242

[59] van der Nest MA, Chavez R, De Vos L, Duong TA, Gil-Duran C, Ferreira MA et al. IMA genome - F14: Draft genome sequences of *Penicillium roqueforti*, *Fusarium sororula*, *Chrysoporthe puriensis*, and *Chalaropsis populi*. IMA Fungus. 2021;12:5. 10.1186/s43008-021-00055-1 PMC7934431 33673862

[60] Marin-Felix Y, Hernandez-Restrepo M, Wingfield MJ, Akulov A, Carnegie AJ, Cheewangkoon R, et al. Genera of phytopathogenic fungi: GOPHY 2. Stud Mycol. 2019;92:47–133. 29997401 10.1016/j.simyco.2018.04.002 PMC6031069

[61] Wingfield BD, De Vos L, Wilson AM, Duong TA, Vaghefi N, Botes A et al. IMA Genome - F16: Draft genome assemblies of *Fusarium marasasianum*, *Huntiella abstrusa*, two *Immersiporthe knoxdaviesiana* isolates, *Macrophomina pseudophaseolina*, *Macrophomina phaseolina*, *Naganishia randhawae*, and *Pseudocercospora cruenta*. IMA Fungus. 2022;13:3. 10.1186/s43008-022-00089-z PMC8867778 35197126

[62] Wilson AM, Gabriel R, Singer SW, Schuerg T, Wilken PM, van der Nest MA, et al. Doing it alone: unisexual reproduction in filamentous ascomycete fungi. Fungal Biol Rev. 2021;35:1–13.

[63] Wingfield BD, Berger DK, Steenkamp ET, Lim HJ, Duong TA, Bluhm BH, et al. IMA Genome-F 8: draft genome of *Cercospora zeina*, *Fusarium pininemorale*, *Hawksworthiomyces lignivorus*, *Huntiella decipiens* and *Ophiostoma Ips*. IMA Fungus. 2017;8:385–96. 29242781 10.5598/imafungus.2017.08.02.10 PMC5729718

[64] van der Nest MA, Beirn LA, Crouch JA, Demers JE, de Beer ZW, De Vos L, et al. IMA Genome-F 3: draft genomes of *A**manita **jacksonii*, *C**eratocystis albifundus*, *F**usarium **circinatum*, *Huntiella omanensis*, *L**eptographium procerum*, *R**utstroemia sydowiana*, and *Sclerotinia echinophila*. IMA Fungus. 2014;5:473–86. 25734036 10.5598/imafungus.2014.05.02.11 PMC4329328

[65] der Van Nest MA, Steenkamp ET, McTaggart AR, Trollip C, Godlonton T, Sauerman E, et al. Saprophytic and pathogenic fungi in the Ceratocystidaceae differ in their ability to metabolize plant-derived sucrose. BMC Evol Biol. 2015;15:273. 26643441 10.1186/s12862-015-0550-7 PMC4672557

[66] Aylward J, Steenkamp ET, Dreyer LL, Roets F, Wingfield BD, Wingfield MJ. Genome sequences of *Knoxdaviesia capensis* and *K. proteae* (Fungi: Ascomycota) from *Protea* trees in South Africa. Stand Genomic Sci. 2016;11:22. 26933475 10.1186/s40793-016-0139-9 PMC4772463

[67] Aylward J, Steenkamp ET, Dreyer LL, Roets F, Wingfield MJ, Wingfield BD. Genetic basis for high population diversity in *Protea*-associated *Knoxdaviesia*. Fungal Genet Biol. 2016;96:47–57. 27720822 10.1016/j.fgb.2016.10.002

[68] Duong TA, Aylward J, Ametrano CG, Poudel B, Santana QC, Wilken PM, et al. IMA Genome - F15: draft genome assembly of *Fusarium pilosicola*, *Meredithiella fracta*, *Niebla homalea*, *Pyrenophora **t**eres* hybrid WAC10721, and *Teratosphaeria viscida*. IMA Fungus. 2021;12:30. 34645521 10.1186/s43008-021-00077-9 PMC8513234

[69] Simão FA, Waterhouse RM, Ioannidis P, Kriventseva EV, Zdobnov EM. Busco: assessing genome assembly and annotation completeness with single-copy orthologs. Bioinformatics. 2015;31:3210–2. 26059717 10.1093/bioinformatics/btv351

[70] Katoh K, Standley DM. Mafft multiple sequence alignment software version 7: improvements in performance and usability. Mol Biol Evol. 2013;30:772–80. 23329690 10.1093/molbev/mst010 PMC3603318

[71] Capella-Gutierrez S, Silla-Martinez JM, Gabaldon T. Trimal: a tool for automated alignment trimming in large-scale phylogenetic analyses. Bioinformatics. 2009;25:1972–3. 19505945 10.1093/bioinformatics/btp348 PMC2712344

[72] Kück P, Longo GC. FASconCAT-G extensive functions for multiple sequence alignment preparations concerning phylogenetic studies. Front Zool. 2014;11:81. 25426157 10.1186/s12983-014-0081-x PMC4243772

[73] Kalyaanamoorthy S, Minh BQ, Wong TKF, von Haeseler A, Jermiin LS. ModelFinder: fast model selection for accurate phylogenetic estimates. Nat Methods. 2017;14:587–9. 28481363 10.1038/nmeth.4285 PMC5453245

[74] Chernomor O, von Haeseler A, Minh BQ. Terrace aware data structure for phylogenomic inference from supermatrices. Syst Biol. 2016;65:997–1008. 27121966 10.1093/sysbio/syw037 PMC5066062

[75] Letunic I, Bork P. Interactive tree of life (iTOL) v5: an online tool for phylogenetic tree display and annotation. Nucleic Acids Res. 2021;49:W293–6. 33885785 10.1093/nar/gkab301 PMC8265157

[76] Martin SH, Wingfield BD, Wingfield MJ, Steenkamp ET. Causes and consequences of variability in peptide mating pheromones of ascomycete fungi. Mol Biol Evol. 2011;28:1987–2003. 21252281 10.1093/molbev/msr022

[77] Keller O, Kollmar M, Stanke M, Waack S. A novel hybrid gene prediction method employing protein multiple sequence alignments. Bioinformatics. 2011;27:757–63. 21216780 10.1093/bioinformatics/btr010

[78] Solovyev V. Statistical approaches in eukaryotic gene prediction. In: Balding DJ, Bishop M, Cannings C, editors. Handbook of Statistical Genetics. John Wiley & Sons: Chichester, UK; 2004. pp. 97–159.

[79] Gasteiger E, Hoogland C, Gattiker A, Wilkins MR, Appel RD, Bairoch A. Protein identification and analysis tools on the expasy server. In: Walker JM, editor. The proteomics protocols handbook. Totowa, USA: Humana; 2005. pp. 571–607.

[80] Käll L, Krogh A, Sonnhammer EL. Advantages of combined transmembrane topology and signal peptide prediction–the Phobius web server. Nucleic Acids Res. 2007;35:W429–32. 17483518 10.1093/nar/gkm256 PMC1933244

[81] Katoh K, Rozewicki J, Yamada KD. MAFFT online service: multiple sequence alignment, interactive sequence choice and visualization. Brief Bioinform. 2019;20:1160–6. 28968734 10.1093/bib/bbx108 PMC6781576

[82] Kuraku S, Zmasek CM, Nishimura O, Katoh K. aLeaves facilitates on-demand exploration of metazoan gene family trees on MAFFT sequence alignment server with enhanced interactivity. Nucleic Acids Res. 2013;41:W22-8. 23677614 10.1093/nar/gkt389 PMC3692103

[83] Gilchrist CLM, Chooi YH. Clinker & clustermap.js: automatic generation of gene cluster comparison figures. Bioinformatics 2021:2473–5. 10.1093/bioinformatics/btab007 33459763

[84] Schmoll M, Seibel C, Tisch D, Dorrer M, Kubicek CP. A novel class of peptide pheromone precursors in ascomycetous fungi. Mol Microbiol. 2010;77:1483–501. 20735770 10.1111/j.1365-2958.2010.07295.x PMC3068285

[85] Witthuhn RC, Harrington TC, Wingfield BD, Steimel JP, Wingfield MJ. Deletion of the *MAT-*2 mating-type gene during uni-directional mating-type switching in *Ceratocystis*. Curr Genet. 2000;38:48–52. 10953881 10.1007/s002940000131

[86] Kile GA, Harrington TC, Yuan ZQ, Dudzinski MJ, Old KM. *Ceratocystis eucalypti* sp. nov., a vascular stain fungus from eucalypts in Australia. Mycol Res. 1996;100:571–9.

[87] Mayers CG, Harrington TC, Masuya H, Jordal BH, McNew DL, Shih HH, et al. Patterns of coevolution between ambrosia beetle mycangia and the *Ceratocystidaceae*, with five new fungal genera and seven new species. Persoonia. 2020;44:41–66. 33116335 10.3767/persoonia.2020.44.02 PMC7567963

[88] Clark NL, Aagaard JE, Swanson WJ. Evolution of reproductive proteins from animals and plants. Reproduction. 2006;131:11–22. 16388004 10.1530/rep.1.00357

[89] Swanson WJ, Vacquier VD. The rapid evolution of reproductive proteins. Nat Rev Genet. 2002;3:137–44. 11836507 10.1038/nrg733

[90] Taylor JW, Turner E, Townsend JP, Dettman JR, Jacobson D. Eukaryotic microbes, species recognition and the geographic limits of species: examples from the kingdom fungi. Philos Trans R Soc Lond B Biol Sci. 2006;361:1947–63. 17062413 10.1098/rstb.2006.1923 PMC1764934

[91] Dignard D, El-Naggar AL, Logue ME, Butler G, Whiteway M. Identification and characterization of *MFA1*, the gene encoding *Candida albicans* a-factor pheromone. Eukaryot Cell. 2007;6:487–94. 17209123 10.1128/EC.00387-06 PMC1828930

[92] Wilson AM, Coetzee MPA, Wingfield MJ, Wingfield BD. Needles in fungal haystacks: discovery of a putative a-factor pheromone and a unique mating strategy in the *L**eotiomycetes*. PLoS One. 2023;18:e0292619. 37824487 10.1371/journal.pone.0292619 PMC10569646

[93] Turner E, Jacobson DJ, Taylor JW. Reinforced postmating reproductive isolation barriers in *Neurospora*, an ascomycete microfungus. J Evol Biol. 2010;23:1642–56. 20546092 10.1111/j.1420-9101.2010.02030.x

[94] van der Walt D, Steenkamp ET, Wingfield BD, Wilken PM. Evidence of biparental mitochondrial inheritance from self-fertile crosses between closely related species of *Ceratocystis*. J Fungi. 2023;9:686. 10.3390/jof9060686 PMC10300817 37367622

[95] Lynn KMT, Wingfield MJ, Oliveira LSS, Alfenas AC, Alfenas RF, Marincowitz S et al. Phylogenetic and population genetic analyses reveal patterns of divergence among isolates in the *Ceratocystis manginecans* complex. bioRxiv. 2025. 10.1002/ece3.73652 PMC13171225 42137546

[96] Fourie A, Wingfield MJ, Wingfield BD, van der Nest MA, Loots MT, Barnes I. Inheritance of phenotypic traits in the progeny of a *Ceratocystis* interspecific cross. Fungal Biol. 2018;122:717–29. 29880206 10.1016/j.funbio.2018.03.001

[97] Elder JF, Turner BJ. Concerted evolution of repetitive DNA. Q Rev Biol. 1995;70:297–320. 7568673 10.1086/419073

[98] Nei M, Rooney AP. Concerted and birth-and-death evolution. Annu Rev Genet. 2005;39:121–52. 16285855 10.1146/annurev.genet.39.073003.112240 PMC1464479

[99] ÓhÉigeartaigh SS, Armisén D, Byrne KP, Wolfe KH. Systematic discovery of unannotated genes in 11 yeast species using a database of orthologous genomic segments. BMC Genomics. 2011;12:1–12. 10.1186/1471-2164-12-377 PMC3161974 21791067

[100] Fávaro LCDL, Araújo WLD, Azevedo JLD, Paccola-Meirelles LD. The biology and potential for genetic research of transposable elements in filamentous fungi. Genet Mol Biol. 2005;28:804–13.

[101] Fouché S, Oggenfuss U, Chanclud E, Croll D. A devil’s bargain with transposable elements in plant pathogens. Trends Genet. 2022;38:222–30. 34489138 10.1016/j.tig.2021.08.005

[102] Gioti A, Mushegian AA, Strandberg R, Stajich JE, Johannesson H. Unidirectional evolutionary transitions in fungal mating systems and the role of transposable elements. Mol Biol Evol. 2012;29:3215–26. 22593224 10.1093/molbev/mss132

[103] Sun S, Yadav V, Billmyre RB, Cuomo CA, Nowrousian M, Wang L, et al. Fungal genome and mating system transitions facilitated by chromosomal translocations involving intercentromeric recombination. PLoS Biol. 2017;15:e2002527. 28800596 10.1371/journal.pbio.2002527 PMC5568439

[104] Galagan JE, Calvo SE, Cuomo C, Ma LJ, Wortman JR, Batzoglou S, et al. Sequencing of *Aspergillus nidulans* and comparative analysis with *A. fumigatus* and *A. oryzae*. Nature. 2005;438:1105–15. 16372000 10.1038/nature04341

[105] Eichler EE, Sankoff D. Structural dynamics of eukaryotic chromosome evolution. Science. 2003;301:793–7. 12907789 10.1126/science.1086132

[106] Bölker M, Kahmann R. Sexual pheromones and mating responses in fungi. Plant Cell. 1993;5:1461–9. 8281042 10.1105/tpc.5.10.1461 PMC160376

[107] Vitale S, Partida-Hanon A, Serrano S, Martinez-Del-Pozo A, Di Pietro A, Turrà D, et al. Structure-activity relationship of α mating pheromone from the fungal pathogen *Fusarium oxysporum*. J Biol Chem. 2017;292:3591–602. 28100777 10.1074/jbc.M116.766311 PMC5339745

[108] Dyer P, Paoletti M, Archer D. Genomics reveals sexual secrets of *Aspergillus*. Microbiology. 2003;149:2301–3. 12949156 10.1099/mic.0.C0119-0

[109] Srikant S, Gaudet R, Murray AW. Extending the reach of homology by using successive computational filters to find yeast pheromone genes. Curr Biol. 2023;33:4098–110. e3. 37699395 10.1016/j.cub.2023.08.039 PMC10592104

[110] Kim H, Borkovich KA. A pheromone receptor gene, *pre-1*, is essential for mating type-specific directional growth and fusion of trichogynes and female fertility in *Neurospora crassa*. Mol Microbiol. 2004;52:1781–98. 15186425 10.1111/j.1365-2958.2004.04096.x

[111] Pöggeler S, Kück U. Comparative analysis of the mating-type loci from *Neurospora crassa* and *Sordaria macrospora*: identification of novel transcribed ORFs. Mol Gen Genet. 2000;263:292–301. 10778748 10.1007/s004380051171

[112] Parada-Rojas CH, Stahr M, Childs KL, Quesada-Ocampo LM. Effector repertoire of the sweetpotato black rot fungal pathogen *Ceratocystis fimbriata*. Mol Plant Microbe Interact. 2024;37:315–26. 38353601 10.1094/MPMI-09-23-0146-FI

[113] Lin JC, Parrish W, Eilers M, Smith SO, Konopka JB. Aromatic residues at the extracellular ends of transmembrane domains 5 and 6 promote ligand activation of the G protein-coupled α-factor receptor. Biochemistry. 2003;42:293–301. 12525156 10.1021/bi026766o

[114] Lee BK, Khare S, Naider F, Becker JM. Identification of residues of the *Saccharomyces cerevisiae* G protein-coupled receptor contributing to α-factor pheromone binding. J Biol Chem. 2001;276:37950–61. 11495900 10.1074/jbc.M103579200

[115] Wilson AM, Lelwala RV, Taylor PWJ, Wingfield MJ, Wingfield BD, Dunlap J. Unique patterns of mating pheromone presence and absence could result in the ambiguous sexual behaviors of *Colletotrichum* species. Volume G3. Genomes: Genes; Genetics. 2021. 10.1093/g3journal/jkab187 PMC8661429 34544120

[116] Merényi Z, Krizsán K, Sahu N, Liu XB, Bálint B, Stajich JE, et al. Genomes of fungi and relatives reveal delayed loss of ancestral gene families and evolution of key fungal traits. Nat Ecol Evol. 2023;7:1221–31. 37349567 10.1038/s41559-023-02095-9 PMC10406608

[117] Harrington T, Aghayeva D, Fraedrich S. New combinations in *Raffaelea*, *Ambrosiella*, and *Hyalorhinocladiella*, and four new species from the redbay ambrosia beetle, *Xyleborus glabratus*. Mycotaxon. 2010;111:337–61.

[118] Turrà D, El Ghalid M, Rossi F, Di Pietro A. Fungal pathogen uses sex pheromone receptor for chemotropic sensing of host plant signals. Nature. 2015;527:521–4. 26503056 10.1038/nature15516

[119] Ramaswe JB, Steenkamp ET, De Vos L, Fru FF, Adegeye OO, Wingfield BD. Sex pheromone receptor Ste2 orchestrates chemotropic growth towards pine root extracts in the pitch canker pathogen *Fusarium circinatum*. Pathogens. 2024;13:425. 38787277 10.3390/pathogens13050425 PMC11124031

[120] Weber RWS, Tribe HT. *Thielaviopsis basicola* and *T. thielavioides*, two ubiquitous moulds on carrots sold in shops. Mycologist. 2004;18:6–10.

[121] Abdullah S, Asensio L, Monfort E, Gomez-Vidal S, Salinas J, Lorca L, et al. Incidence of the two date palm pathogens, *Thielaviopsis paradoxa* and *T. punctulata* in soil from date palm plantations in Elx, South-East Spain. J Plant Prot Res. 2009;49:276–9.

[122] Heller WP, Harrington TC, Brill E, Keith LM. High-sensitivity ITS real-time PCR assays for detection of *Ceratocystis lukuohia* and *Ceratocystis huliohia* in soil and air samples. PhytoFront. 2023;3:148–55.

[123] Loppnau P, Tanguay P, Breuil C. Isolation and disruption of the melanin pathway polyketide synthase gene of the softwood deep stain fungus *Ceratocystis resinifera*. Fungal Genet Biol. 2004;41:33–41. 14643257 10.1016/j.fgb.2003.08.008

[124] Tzima AK, Paplomatas EJ, Schoina C, Domazakis E, Kang S, Goodwin PH. Successful *Agrobacterium* mediated transformation of *Thielaviopsis basicola* by optimizing multiple conditions. Fungal Biol. 2014;118:675–82. 25110130 10.1016/j.funbio.2014.04.009

[125] Niu X, Pei M, Liang C, Lv Y, Wu X, Zhang R, et al. Genetic transformation and green fluorescent protein labeling in *Ceratocystis paradoxa* from coconut. Int J Mol Sci. 2019;20:2387. 31091742 10.3390/ijms20102387 PMC6566578

[126] Sayari M, van der Nest MA, Steenkamp ET, Adegeye OO, Marincowitz S, Wingfield BD. *Agrobacterium*-mediated transformation of *Ceratocystis albifundus*. Microbiol Res. 2019;226:55–64. 31284945 10.1016/j.micres.2019.05.004

[127] Wilson AM, Wilken PM, van der Nest MA, Wingfield MJ, Wingfield BD. The novel *Huntiella omanensi*s mating gene, *MAT1-2-7*, is essential for ascomatal maturation. Fungal Genet Biol. 2020;137:103335. 31958567 10.1016/j.fgb.2020.103335

[128] Lane FA, Du Plessis D, Wingfield BD, Wilken PM. Transferring an *Agrobacterium*-mediated transformation protocol across eight genera in the Ceratocystidaceae. For Pathol. 2021;51:e12688.

