# Supplementary material for: Lineage-specific diversity of pheromone response pathway genes is independent of mating strategy in Ceratocystidaceae
Source: BMC Genomics. 2026 Feb 23;27:320. doi: 10.1186/s12864-026-12527-y (PMC13037118; doi:10.1186/s12864-026-12527-y)
Supplement: Supplementary file 6 — Supplementary Material 6. Treatment of predicted pheromone receptors lacking seven transmembrane domains. [file 12864_2026_12527_MOESM6_ESM.pdf]

Supplementary File 1: Treatment of predicted pheromone receptors lacking seven transmembrane domains

Where fewer than seven domains were predicted in the pheromone-receptor translations, FGENESH+ (using the generic *Fusarium* gene models) was used to explore alternative gene models guided by the relevant protein sequence of a close relative that had all seven domains. Alternatively, the gene model was compared to the close relative and attempts were made to manually adjust the start codons, stop codons and/or intron boundaries to provide a more plausible gene prediction. Where small indels resulted in a truncated pheromone-receptor gene, these were investigated manually by mapping raw reads (when available) of the relevant genome assembly to the pheromone-receptor locus in CLC Genomics Workbench (v. 22.0), and, if necessary, correcting assembly errors using a reference assembly approach.
