# Supplementary material for: Lineage-specific diversity of pheromone response pathway genes is independent of mating strategy in Ceratocystidaceae
Source: BMC Genomics. 2026 Feb 23;27:320. doi: 10.1186/s12864-026-12527-y (PMC13037118; doi:10.1186/s12864-026-12527-y)
Supplement: Supplementary file 7 — Supplementary Material 7. Explanation for ambiguity in mature α-pheromone peptide numbers. [file 12864_2026_12527_MOESM7_ESM.pdf]

Supplementary File 2: Explanation for ambiguity in mature α-pheromone peptide numbers

There was some ambiguity in the number of mature α-pheromone peptides in *Chalaropsis thielavioides* and *Davidsoniella australis* (Fig. 2). An assembly gap of 100 bp represented by a string of N-base nucleotides was present in the α-pheromone gene of *Ch. thielavioides*. If this represents a true fragment of DNA, it would result in 17 aa that form part of the encoded protein and perhaps a single 11 aa mature peptide, however, this could not be confirmed through assembly of the raw genome sequencing reads (SRA accession DRS023903) due to the highly repetitive sequence. The α-pheromone gene in *D. australis* was at the end of a contig, resulting in a 3’ end truncation and it was not possible to join contigs *in silico* using in-house raw genome sequencing reads.
