# Supplementary material for: Lineage-specific diversity of pheromone response pathway genes is independent of mating strategy in Ceratocystidaceae
Source: BMC Genomics. 2026 Feb 23;27:320. doi: 10.1186/s12864-026-12527-y (PMC13037118; doi:10.1186/s12864-026-12527-y)
Supplement: Supplementary file 8 — Supplementary Material 8. Genes found in the family-conserved α-pheromone locus of Berkeleyomyces species and variation in one Be. rouxiae genome assembly. [file 12864_2026_12527_MOESM8_ESM.pdf]

Supplementary File 3: Genes found in the family-conserved α-pheromone locus of *Berkeleyomyces* species and variation in one *Be. rouxiae* genome assembly

In the *Be. basicola* genome assembly, the region between the monocarboxylate transporter 3 gene and the DUF1640-domain-containing protein encoding gene (where the α-pheromone gene was found in the related species) contained a reverse transcriptase gene. In *Be. rouxiae*, the comparable region contained four genes: a putative double-stranded RNA/RNA-DNA hybrid binding protein gene, a putative gene, a non-LTR retrotransposon, a non-LTR retrovirus reverse gene and a RT-like reverse transcriptase gene. Where two α-pheromone genes were identified on the same contig in *Be. rouxiae* isolate PM34, four truncated copies of the gene were detected in the genome sequence of *Be. rouxiae* LT1, of which three were at the ends of contigs, and a single one which was present on the entirety of a 310 bp contig.
