# Supplementary material for: Lineage-specific diversity of pheromone response pathway genes is independent of mating strategy in Ceratocystidaceae
Source: BMC Genomics. 2026 Feb 23;27:320. doi: 10.1186/s12864-026-12527-y (PMC13037118; doi:10.1186/s12864-026-12527-y)
Supplement: Supplementary file 9 — Supplementary Material 9. Details on additional a-pheromone loci identified in Ceratocystis species. [file 12864_2026_12527_MOESM9_ESM.pdf]

Supplementary File 4: Details on additional a-pheromone loci identified in *Ceratocystis* species

An additional a-pheromone locus (to the family-conserved locus) with a single copy of the a-pheromone gene was present in both *Ce. cacaofunesta* and *Ce. platani*, and was flanked by a diatom spindle kinesin-1 gene and hypothetical gene 9 (Suppl. Fig. 5). This locus was also present in *Ce. colombiana*, *Ce. eucalypticola*, *Ce. fimbriata*, *Ce. lukuohia* and *Ce. manginecans*, although these species had between three and five additional loci in total. Among these additional loci, one locus was clearly shared containing a single a-pheromone gene flanked by hypothetical genes 3 and 9, although in *Ce. colombiana* it was flanked by two copies of the hypothetical gene 9. *Ce. fimbriata* had the most a-pheromone loci with seven unique loci in total, although at most five of these were present in a single genome assembly (isolate LPF 1912), while in another (isolate NC 236), only two loci were present.
