# Supplementary material for: Lineage-specific diversity of pheromone response pathway genes is independent of mating strategy in Ceratocystidaceae
Source: BMC Genomics. 2026 Feb 23;27:320. doi: 10.1186/s12864-026-12527-y (PMC13037118; doi:10.1186/s12864-026-12527-y)
Supplement: Supplementary file 11 — Supplementary Material 11. Supplementary Table 2: GenBank accession numbers for the pheromone-receptor proteins used as query sequences to identify homologs in the Ceratocystidaceae genome assemblies. Supplementary Table 3: Proteins involved in the pheromone response pathway that were used to identify homologs in genome assemblies investigated. Supplementary Table 4: Consolidation of findings to identify any correlation between mating strategy and pheromone-receptor system in Ceratocystidaceae species. [file 12864_2026_12527_MOESM11_ESM.docx]

### Supplementary Table 2: GenBank accession numbers for the pheromone-receptor proteins used as query sequences to identify homologs in the *Ceratocystidaceae* genome assemblies

| **Species** | **a-pheromone receptor** | **α-pheromone receptor** |
| --- | --- | --- |
| *Fusarium graminearum* | PCD40677 | N/A |
| *F. oxysporum* | SCO83188 | EWZ38561 |
| *F. subglutinans* | N/A | KAF5614212 |
| *Trichoderma harzianum* | KKP06116 | KKP04616 |
| *Tr. reesei* | AEU12196 | EGR47492 |

### Supplementary Table 3: Proteins involved in the pheromone response pathway that were used to identify homologs in genome assemblies investigated

| **Protein** | **Description** | **Species** | **Accession number** |
| --- | --- | --- | --- |
| **Processing of the α-pheromone protein** | | | |
| KEX1 | Pheromone-processing carboxypeptidase | *Aspergillus niger* | GJP98016 |
| KEX2 | Pheromone-processing endoprotease | *Aspergillus niger* | GJP95054 |
| STE13 | Dipeptidyl aminopeptidase | *Aspergillus nidulans* | DAA01787 |
| **Processing of the a-pheromone protein** | | | |
| RAM1 | Protein farnesyltransferase | *Aspergillus niger* | GJP87387 |
| RAM2 | Protein farnesyltransferase | *Aspergillus niger* | GJP87721 |
| RCE1 | CAAX prenyl protease | *Aspergillus niger* | GJP93700 |
| STE6 | ATP-binding cassette a-factor transporter | *Aspergillus luchuensis* | GAT25785 |
| STE14 | Farnesyl cysteine-carboxyl methyltransferase | *Aspergillus niger* | GJP89046 |
| STE24 | CAAX prenyl protease | *Aspergillus niger* | GAQ37481 |
| AXL1 | Endoprotease | *Aspergillus niger* | DAA35002 |
| **Signal transduction pathway** | | | |
| GNA1 | G-protein alpha-1 subunit | *Aspergillus niger* | GJP88677 |
| GNA2 | G-protein alpha-2 subunit | *Aspergillus nidulans* | AAD34893 |
| GNB1 | G-protein beta subunit | *Aspergillus niger* | RDH24828 |
| GNG1 | G-protein gamma subunit | *Aspergillus luchuensis* | XP_041539406 |
| MEKK3 ^*^ | Mitogen activated protein kinase kinase kinase | *Aspergillus niger* | GAQ36387 |
| MEK2 | Mitogen activated protein kinase kinase 2 | *Aspergillus niger* | GAQ44310 |
| MAK2 | Mitogen-activated protein kinase-2 | *Aspergillus niger* | GAQ45255 |
| PP1 | Serine/threonine-protein phosphatase | *Aspergillus niger* | GAQ38943 |

* Also referred to as NRC1

Supplementary Table 4: Consolidation of findings to identify any correlation between mating strategy and pheromone-receptor system in *Ceratocystidaceae* species

|  | **Heterothallism**  **(17 species)** | **Primary homothallism**  **(4 species)** | **Unisexuality**  **(3 species)** | **Mating-type switching**  **(19 species)** |
| --- | --- | --- | --- | --- |
| **α-pheromone gene** | | | | |
| Locus position (%) | 88 | N/A^2^ | 100 | 100 |
| No. of mature peptides encoded per gene | 1 - 13 | 2^2^ | 4 - 6 | 1 - 4 |
| Mature peptide size (aa) | 10 - 11 | 10^2^ | 11 | 10 - 11 |
| No. of gene copies | 1 - 2 | 1^2^ | 1 | 1 |
| **α-pheromone-receptor gene** | | | | |
| Locus position (%) | 65 | 75 | 100 | 100 |
| Number of species encoding seven transmembrane domains (%) | 100 | 25 | 100 | 100 |
| Protein size (aa) | 357 - 401 | 228 - 398 | 401 | 334 - 403 |
| **a-pheromone gene** | | | | |
| Locus position (%) | 88 | 100 | 100 | 100 |
| No. of mature peptides encoded per gene | 1 - 5 | 1 | 1 - 2^3^ | 1 - 3 |
| Mature peptide size (aa) | 8 - 10 | 8 | 9 | 8 - 10 |
| No. of gene copies | 1 - 7 | 1 | 1 - 4^3^ | 1 - 5 |
| **a-pheromone-receptor gene** | | | | |
| Locus position (%) | 100 | 100 | 100 | 100 |
| Number of species encoding seven transmembrane domains (%) | 94 | 100 | 100 | 95 |
| Protein size (aa) | 468 - 542 | 481 - 520 | 493 | 449 - 538 |

^1^ The number of species per mating strategy (presented as percentage) that shared genomic position based on the presence of at least one conserved flanking gene.

^2^ Only includes *P. hubbardii* as *Ambrosiella* species lack this gene.

^3^ Excluding genes with early stop codons.
