# Supplementary material for: Lineage-specific diversity of pheromone response pathway genes is independent of mating strategy in Ceratocystidaceae
Source: BMC Genomics. 2026 Feb 23;27:320. doi: 10.1186/s12864-026-12527-y (PMC13037118; doi:10.1186/s12864-026-12527-y)
Supplement: Supplementary file 12 — Supplementary Material 12. Supplementary Figure 1: Alignments of the pheromone-receptor proteins from species lacking all seven transmembrane domains with pheromone-receptor proteins from species within the same clade where all seven domains were predicted. (A) The a-pheromone receptor of Br. fagacearum compared with P. hubbardiid and W. franznegeri. (B) The a-pheromone receptor of To. taiwanensis aligned to Ambrosiella proteins. (C) The α-pheromone receptor of Ambrosiella aligned to that of To. taiwanensis. Predicted extracellular regions are indicated by yellow rectangles, cytoplasmic regions by purple and transmembrane domains by blue. Black blocks highlight regions where transmembrane domains are absent or adjacent domains may have merged into one. Supplementary Figure 2: A pairwise comparison showing the percentage identity of the α-pheromone receptor proteins between members of a genus. Supplementary Figure 3: A pairwise comparison showing the percentage identity of the a-pheromone receptor proteins between members of a genus. Supplementary Figure 4: A pairwise comparison showing the percentage identity of the mature α-pheromone peptides between members of a genus. Supplementary Figure 5: An alignment of the putative a- and α-pheromone mature peptides. Species are numbered according to their order from top to bottom on the phylogeny (Fig. 1). Supplementary Figure 6: A pairwise comparison showing the percentage identity of the mature a-pheromone peptides between members of a genus. [file 12864_2026_12527_MOESM12_ESM.docx]

**
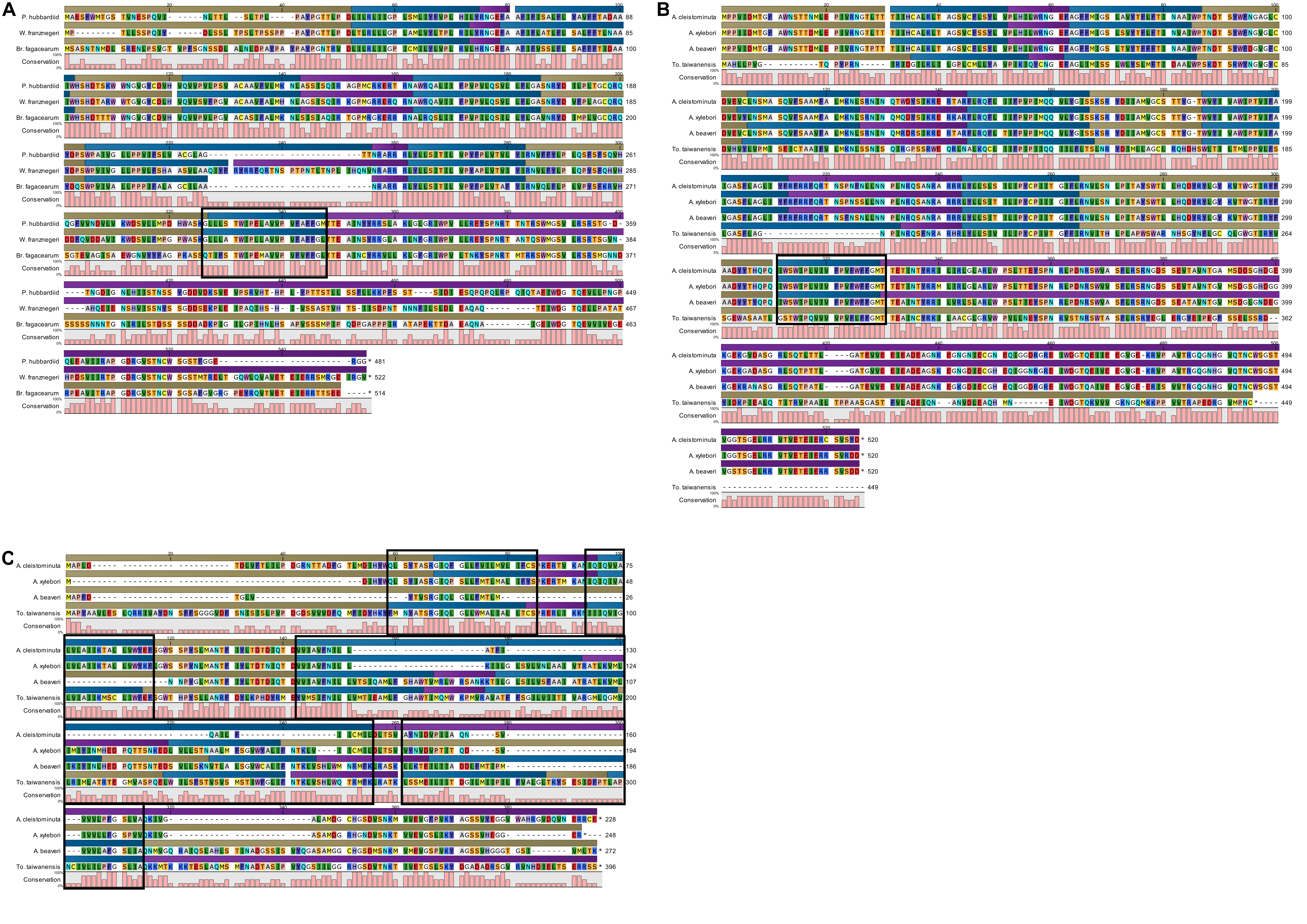
Supplementary Figure 1:** Alignments of the pheromone-receptor proteins from species lacking all seven transmembrane domains with pheromone-receptor proteins from species within the same clade where all seven domains were predicted. (A) The a-pheromone receptor of *Br. fagacearum* compared with *P. hubbardiid* and *W. franznegeri*. (B) The a-pheromone receptor of *To. taiwanensis* aligned to *Ambrosiella* proteins. (C) The α-pheromone receptor of *Ambrosiella* aligned to that of *To. taiwanensis*. Predicted extracellular regions are indicated by yellow rectangles, cytoplasmic regions by purple and transmembrane domains by blue. Black blocks highlight regions where transmembrane domains are absent or adjacent domains may have merged into one.


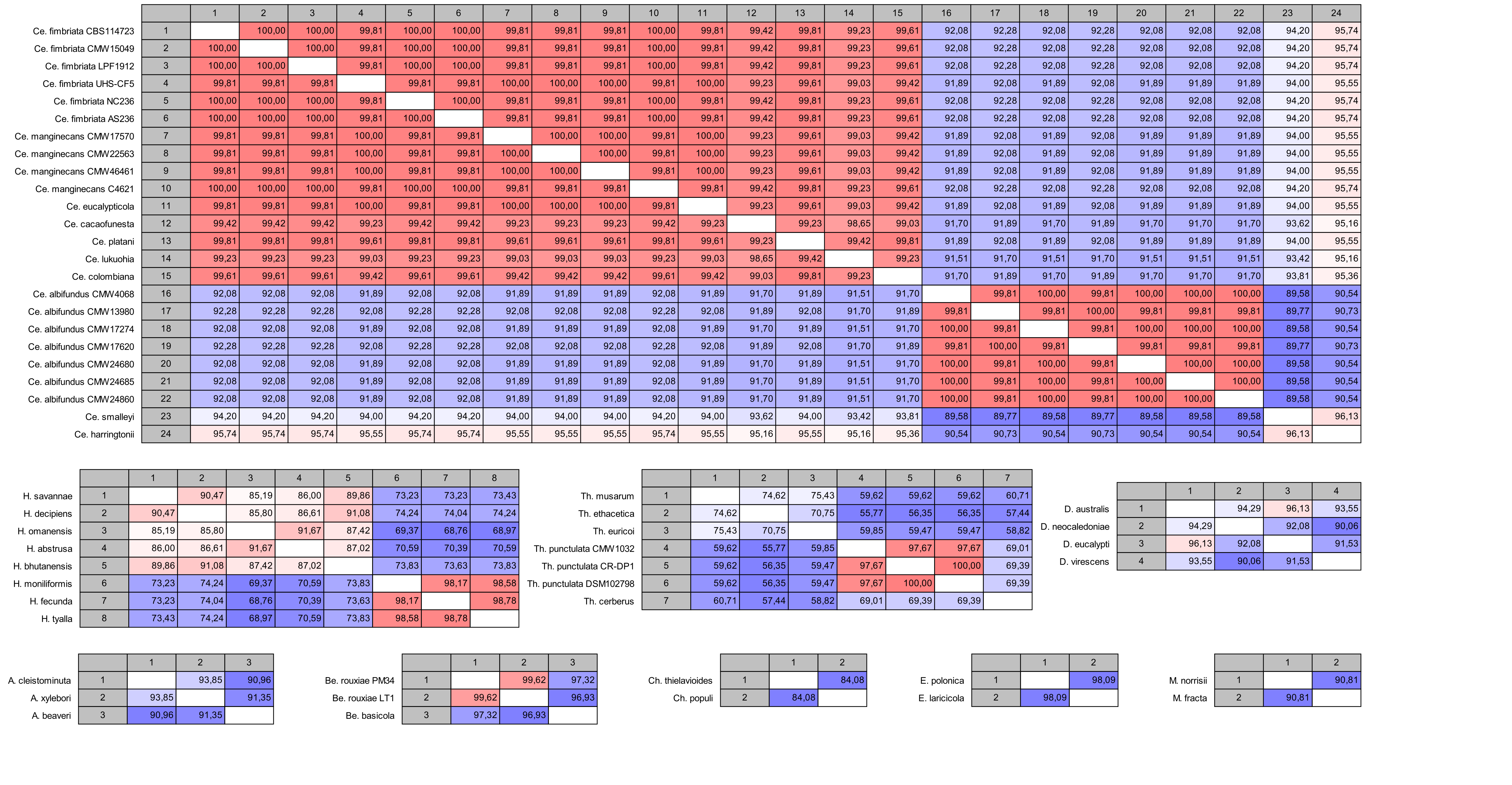
**Supplementary Figure 2:** A pairwise comparison showing the percentage identity of the α-pheromone receptor proteins between members of a genus.


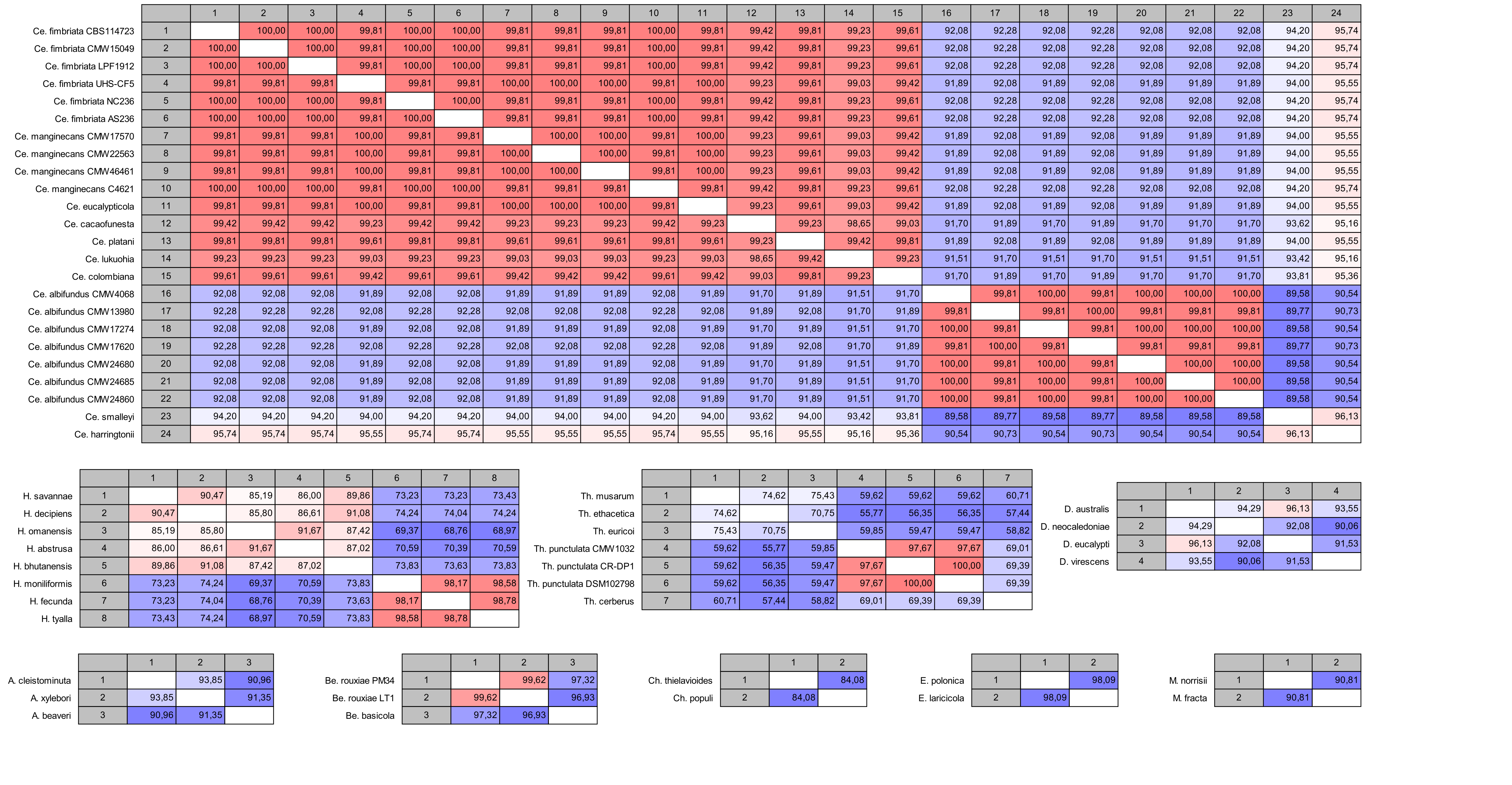
**Supplementary Figure 3:** A pairwise comparison showing the percentage identity of the a-pheromone receptor proteins between members of a genus.

*
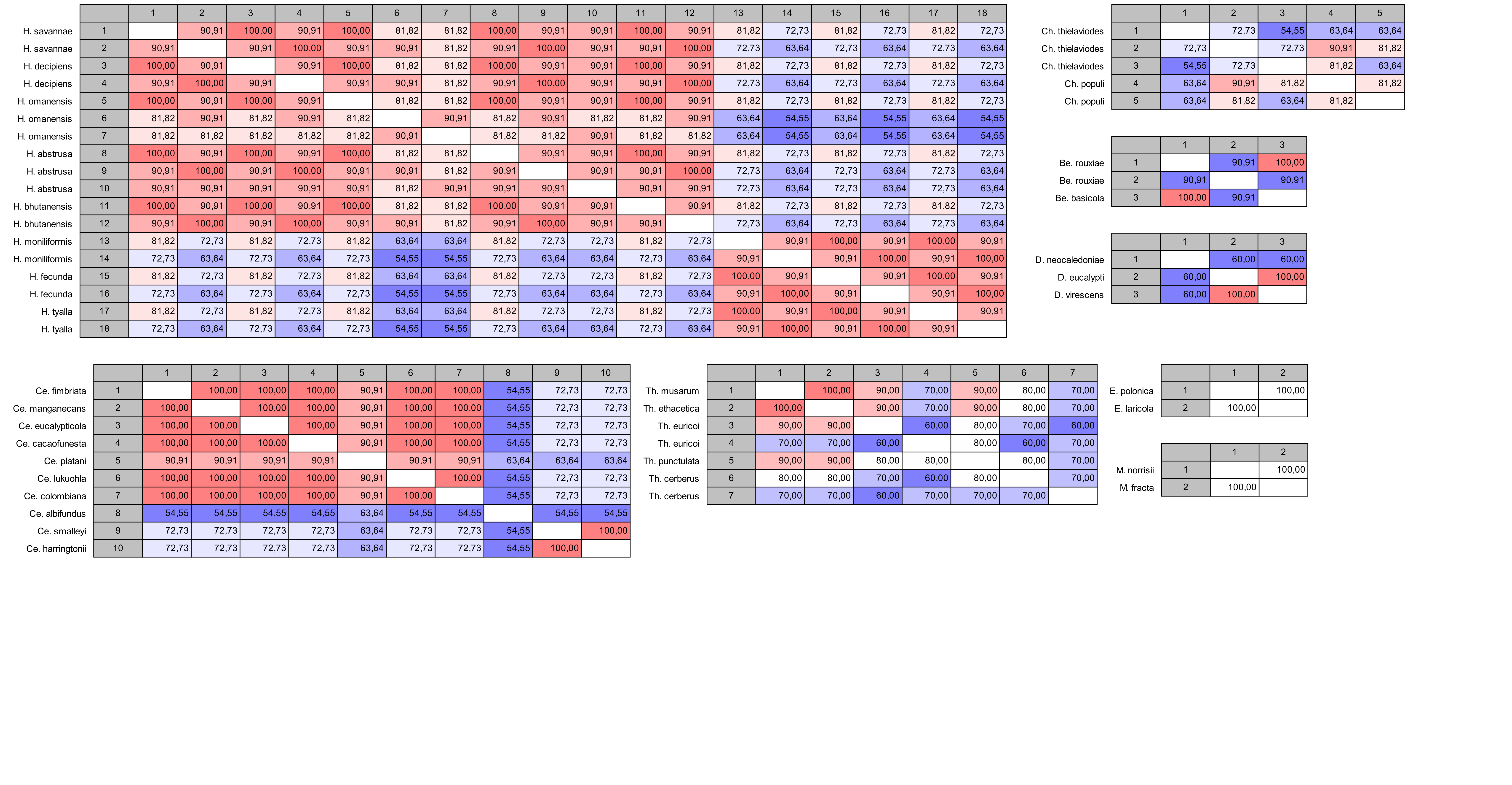
***Supplementary Figure 4:** A pairwise comparison showing the percentage identity of the mature α-pheromone peptides between members of a genus.

**
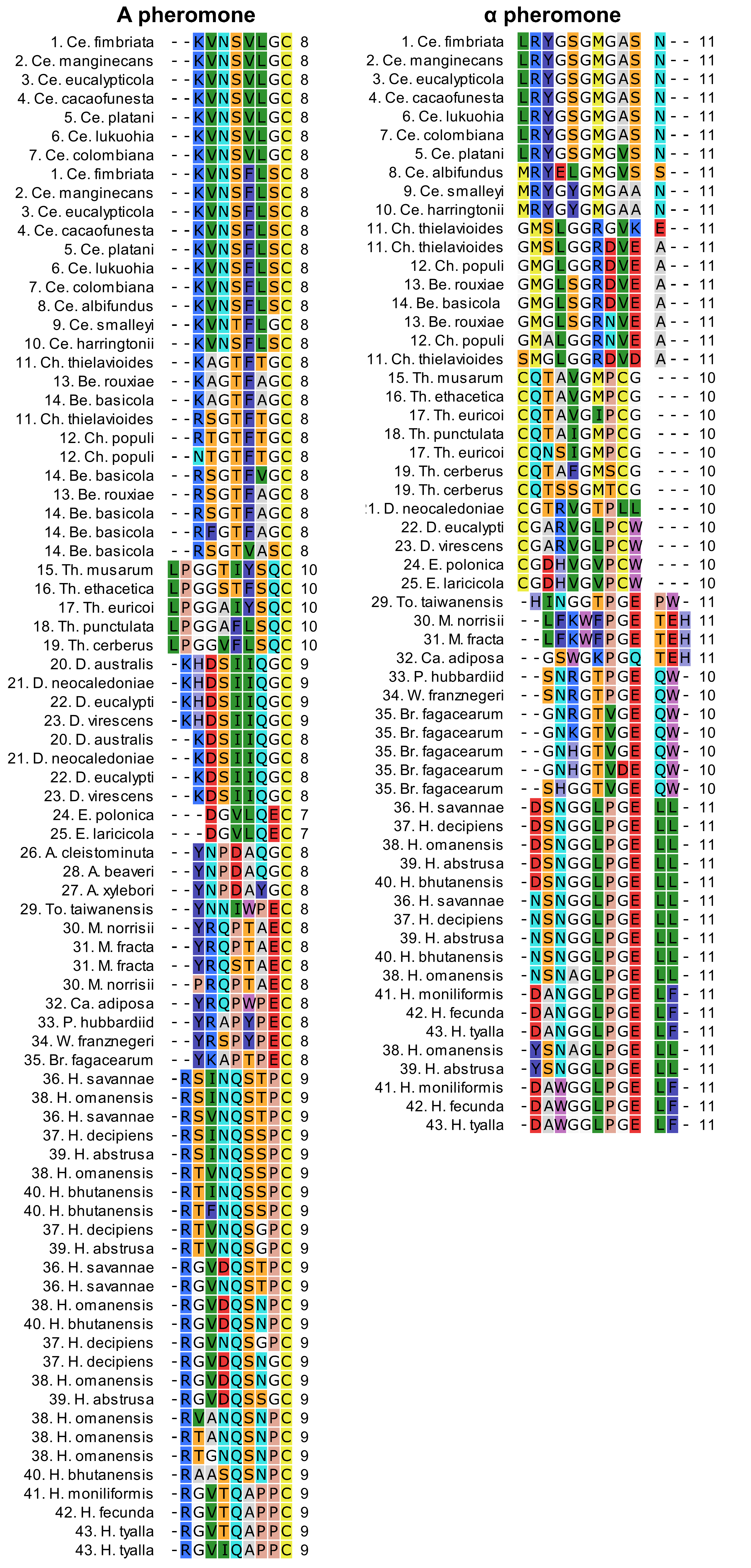
**

**Supplementary Figure 5:** An alignment of the putative a- and α-pheromone mature peptides. Species are numbered according to their order from top to bottom on the phylogeny (Fig. 1).


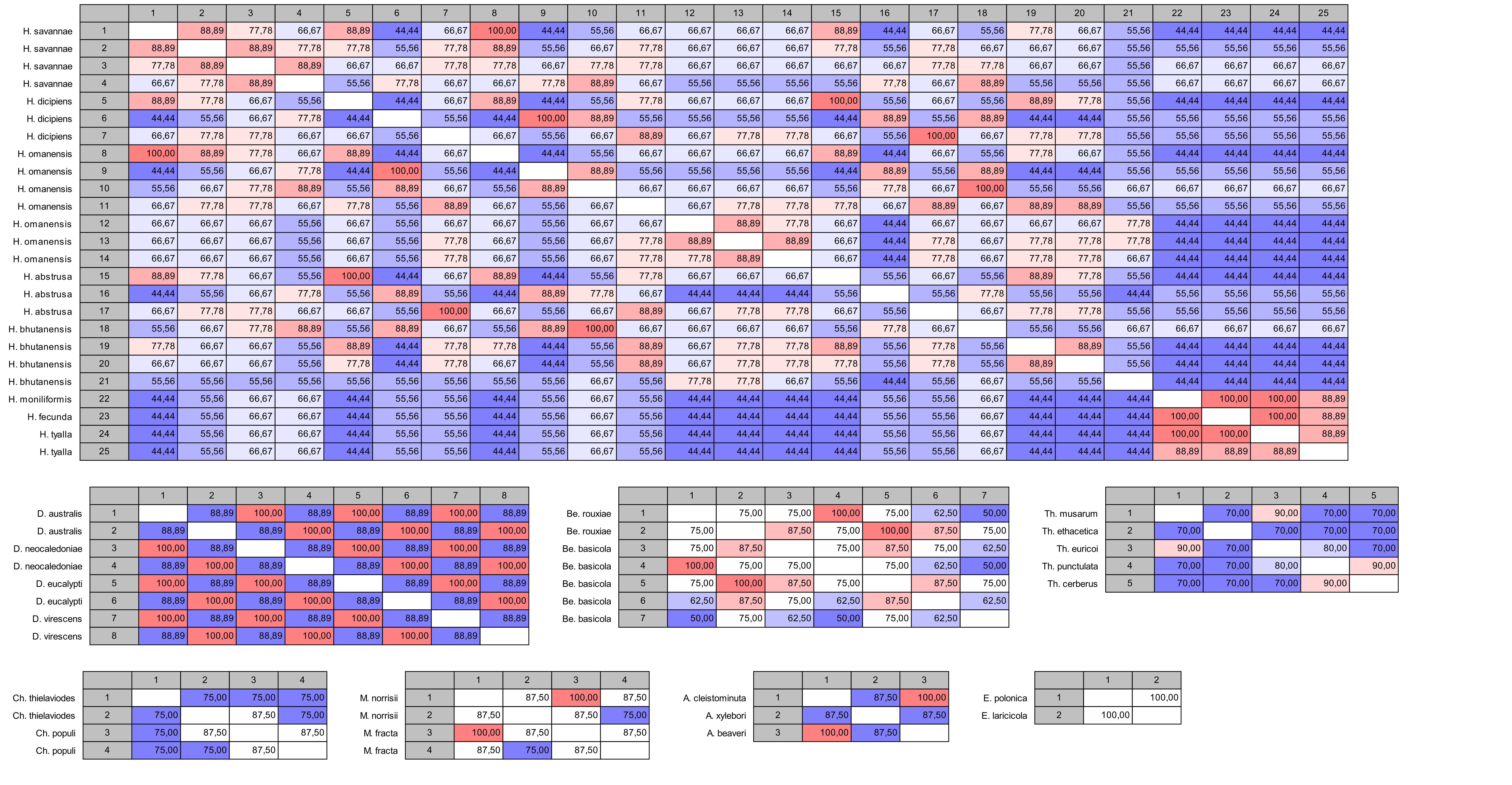


**
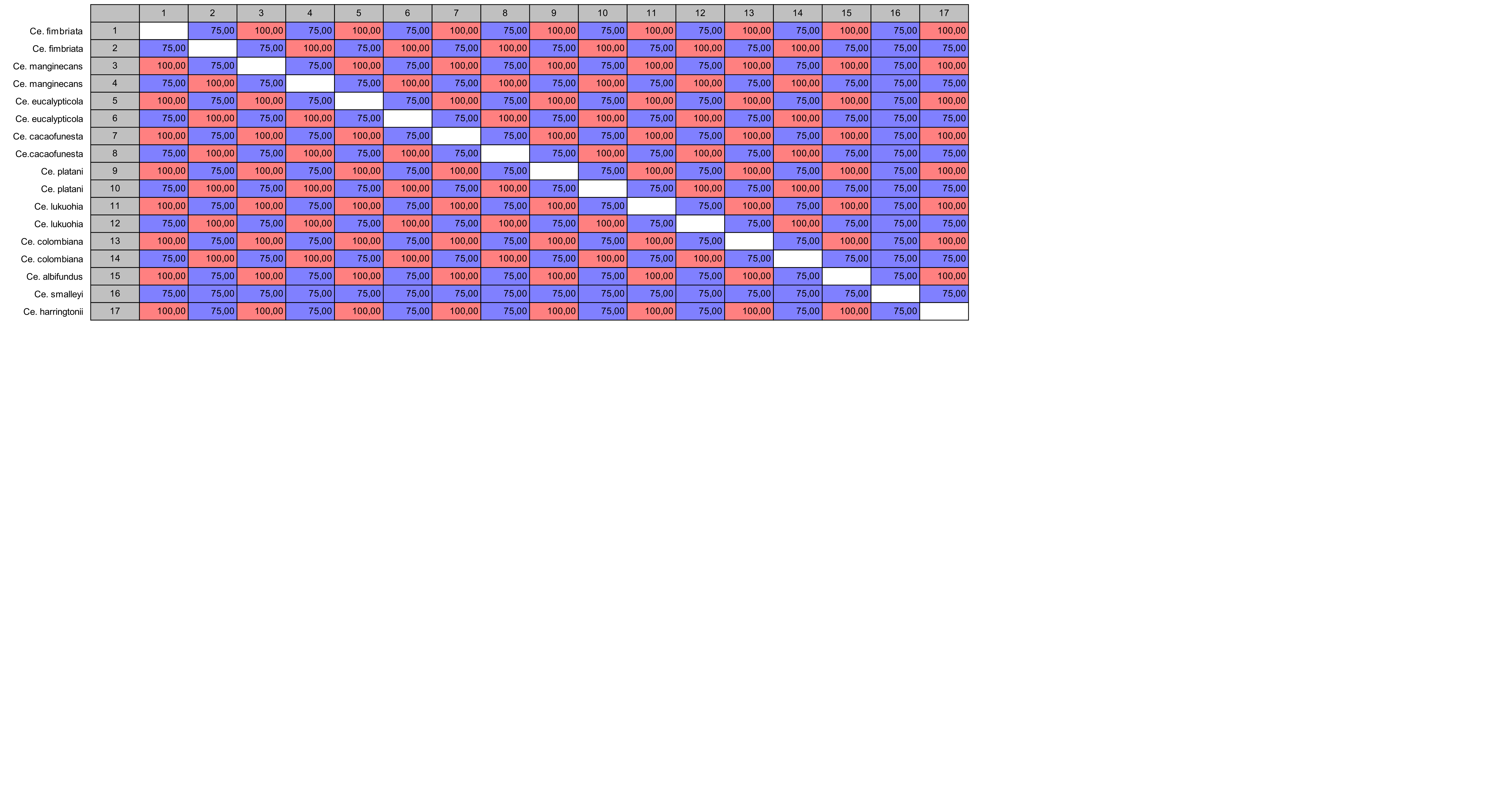
Supplementary Figure 6:** A pairwise comparison showing the percentage identity of the mature a-pheromone peptides between members of a genus.





**Supplementary Figure 7:** Two additional a-pheromone loci. “Hypothetical” genes were named as such if a BLAST result was to a protein listed as “hypothetical” on NCBI, and these genes were numbered based on their order of appearance across all the various a-pheromone loci in the different species. Genes that did not have homology to any other genes in the dataset are indicated as grey arrows.


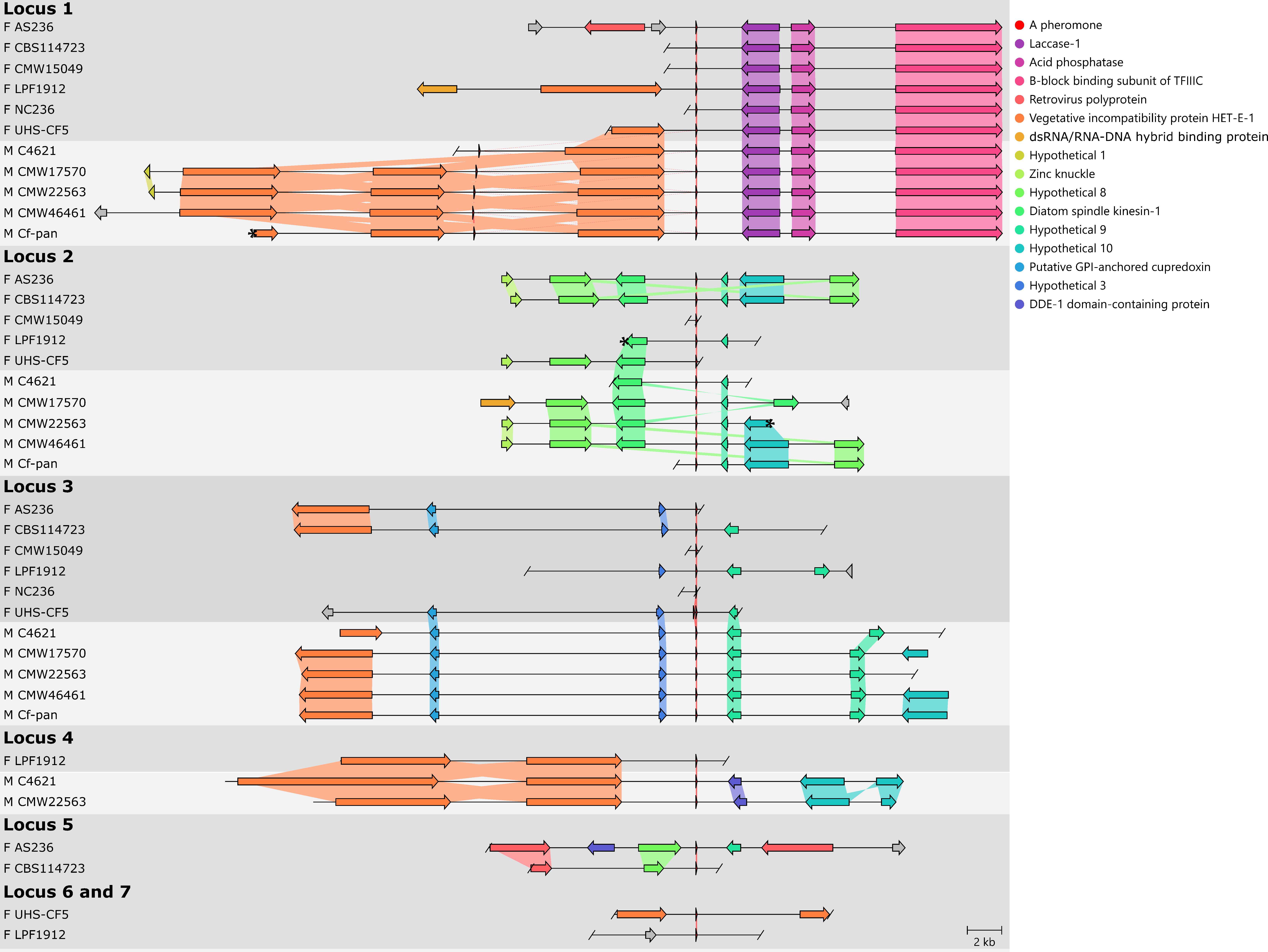


**Supplementary Figure 8:** Intraspecies variation observed between the flanking genes of homologous a-pheromone loci. F represents *Ce. fimbriata* isolates while M represents *Ce. manginecans* isolates. End of contigs were indicated with a slash or an asterisk when the end of the of a contig is within a gene. “Hypothetical” genes were named as such if a BLAST result was to a protein listed as “hypothetical” on NCBI, while “putative gene” were genes that were predicted but had no valid BLAST result. These genes were numbered based on their order of appearance in the figure.
